# Multivariate Optimization of Self-doped TiO_2_ Nanotube Synthesis for Enhanced Optical and Electronic Performance

**DOI:** 10.1021/acsomega.6c08217

**Published:** 2026-09-28

**Authors:** João Vitor Martins, Gabrielle Sarto, Thiago Nogueira Marques Cervantes, Paulo Rogério Catarini da Silva, Lucio Cesar de Almeida

**Affiliations:** † Department of Chemistry, Laboratory of Environmental Electrochemistry (LabEA), State University of Londrina (UEL), Celso Garcia Cid Highway, PR 445, km 380, Londrina 86055-900, Brazil; ‡ Department of Physics, Laboratory of Thin Films and Materials (FILMAT), State University of Londrina (UEL), Celso Garcia Cid Highway, PR 445, km 380, Londrina 86055-900, Brazil; § National Institute of Alternative Technologies for Detection, Toxicological Assessment and Removal of Emerging and Radioactive Contaminants—(INCT-Datrem), Rua Professor Francisco Degni, 55, 14800-060 Araraquara, São Paulo, Brazil

## Abstract

This paper presents the cathodic polarization of anodic TiO_2_ nanotube layers (TNT) on Ti to promote the electrochemical reduction of Ti^4+^ lattice into Ti^3+^, accompanied by oxygen-vacancy formation and proton intercalation, resulting in self-doped nanotubes (SD-TNT). Although this reduction route has been known since the early reports of TNT, the quantitative relationship between the polarization parameters and the resulting electronic response remains to be established. Hence, a full factorial design (FFD) coupled to response surface methodology (RSM) was employed to assess simultaneously the cathodic potential, the polarization (self-doping) time, and the calcination temperature, using the cathodic current density (*j*_c_) associated with the Ti^4+^/Ti^3+^ reduction as the response. The optimum condition, obtained by FFD/RSM, was −1.5 V, applied for 480 min, to layers calcined at 450 °C, which delivered a *j*_c_ of −0.325 mA cm^–2^. Under these conditions, SD-TNT were also investigated using electrochemical impedance spectroscopy (EIS), which revealed a decrease of the charge-transfer resistance from 831.9 to 92.35 Ω, together with a lower capacitance, constant phase element exponent, and a shorter time constant, consistent with a higher density of Ti^3+^ centers and oxygen vacancies distributed along the tube axis. Raman deconvolution of the anatase E_g_ mode confirmed the increase of the oxygen-vacancy contribution after cathodic polarization, whereas the calcination temperature controlled the anatase/rutile ratio and, therefore, the apparent band gap. The multivariate approach, thus, provides quantitative criteria to select the cathodic polarization parameters of TNT.

## Graphical abstract

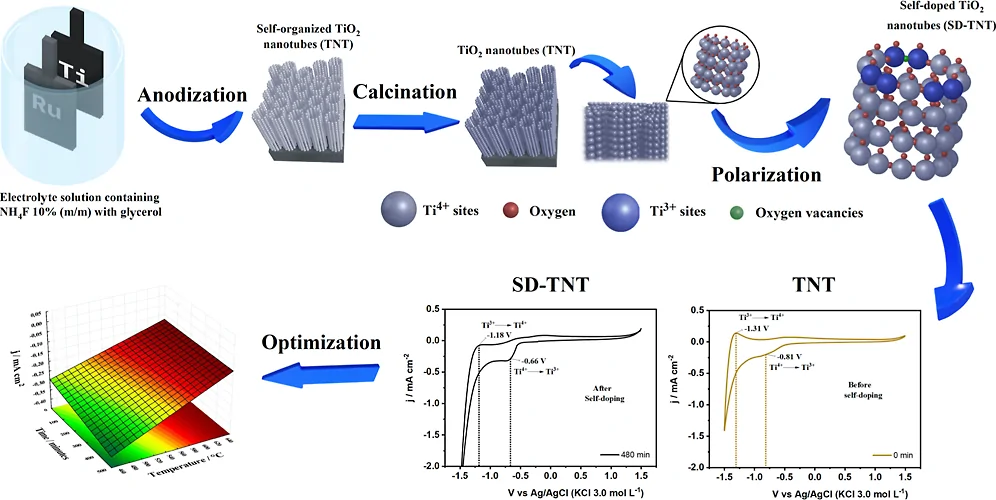

## 1 Introduction

Titanium dioxide (TiO_2_) is a multifunctional semiconductor material with high biocompatibility, low toxicity, chemical and mechanical stability, and photocatalytic activity.^1–6^ These characteristics make this material an interesting and promising alternative for a wide variety of applications, including a platform for the remediation of recalcitrant contaminants,^7–9^ photoanode for dye-sensitized solar cells (DSSCs),^10,11^ whose overall device performance also depends on the photoanode surface engineering and the counter-electrode architecture,^12,13^ energy conversion and storage,^14^ supercapacitor devices,^15–20^ and sensorial platforms.^21–23^ The potential applicability of TiO_2_ films may depend considerably on the crystallinity of this material.^24,25^

Multiple optimization approaches have been implemented to improve the functional properties of nanostructured TiO_2_ films. One of the most widely used strategies involves applying it at the nanoscale in different morphologies, such as nanoparticles, nanosheets, and nanotubes.^24,26–28^ These nanostructured materials exhibit a substantially larger surface area than the conventional materials, demonstrating a marked increase in response gains (e.g., catalytic, photocatalytic), attributed to the higher density of potential adsorption sites, which consequently intensifies interactions at the interfacial region.^24,25^ Among the various morphologies of TiO_2_ synthesized, TiO_2_ nanotubes (TNT) have been extensively evaluated as a photoanode, due to their high surface area, a simple synthesis method, and highly ordered structures, enabling precise control over the TNT length and diameter by adjusting the synthesis time and the applied anodic overpotential, respectively.^29–31^ Furthermore, the heat treatment of the synthesized TNTs has a pivotal effect on this crystallinity, which can impact directly its photoactivity (i.e., in the generation of pairs electron/hole), due in part to the significant difference between the band gap values for the crystalline phases of TiO_2_ (3.0 and 3.2 eV for rutile and anatase, respectively).^9,32,33^ These band gap values are relatively high, requiring radiation energies in the UV region of the spectra to effectively produce electron–hole pairs (i.e., to promote electrons from the valence band to the conduction band).

To shift the required radiation to produce electron–hole pairs to longer wavelengths and consequently the band gap value of TiO_2_, several strategies have been employed, such as a combination of different proportions of the crystalline phases’ anatase and rutile,^33–35^ decoration in the lattice with nanoparticles (e.g., Pt, Ag, Au),^36–38^ heterojunctions by using different metal oxides,^39,40^ and, more recently, the self-doping (e.g., cathodic polarization).^7,8,23,41^ However, considering an electrochemical point of view, TNT shows a highly capacitive behavior, limiting its applicability as an electrode material. As previously mentioned, a sustainable alternative has been widely investigated and applied to overcome this obstacle: a process based on the cathodic polarization of TNT films (known as self-doping).^7,8,23,41,42^ This electrochemical self-doping of TNT films involves the reduction of surface ions Ti^4+^ to Ti^3+^, promoting the formation of oxygen vacancies and proton intercalation on the surface of the TNT crystal lattice, according to the reaction Ti^4+^ + H^+^ + e^–^ → Ti^3+^H^+^, generating the self-doped TNT (SD-TNT). The self-doping process of TNT films (anatase phase) significantly enhances conductivity and alters the electrochemical behavior of SD-TNT compared to the undoped one (i.e., pristine TNT). This improvement is ascribed to the formation of Ti^3+^ centers during the self-doping process,^41–43^ which narrows the material’s band gap and consequently increases its conductivity.^33,41,44^

The electrochemical and thermal reduction of anodic TNT layers is not a recent development and a comprehensive account of its history is required to place the present contribution in context. Already in 2006, Ghicov et al. demonstrated that anodic TNT layers can be reversibly reduced by H^+^ insertion, with pronounced electrochromic effects,^45^ and that the annealing atmosphere governs both the crystallinity and the photoresponse, since annealing under reducing (Ar) atmospheres yields partially reduced, vacancy-rich layers with a lower photocurrent efficiency.^46^ The first deliberate use of electrochemical self-doping to switch the conductivity of TNT layers was reported by Macak et al., who reduced the tube bottoms from Ti^4+^ to Ti^3+^ and observed a decrease of the solid-state resistance by several orders of magnitude, from approximately 70 kΩ to 10 Ω, along with a narrowing of the mobility gap from about 3.2 to 2.4 eV in the reduced state.^47^ The influence of the annealing conditions on the electronic conductivity of anodic TNT layers was subsequently quantified by Tighineanu et al.^48^ Most of the later reports on black, or more accurately reduced, TNT layers rely on annealing under H_2_, frequently at atmospheric pressure, as demonstrated for hydrogen-treated TiO_2_ nanowires and nanotubes,^19,49^ by hydrogen plasma treatment^50^ and by high-pressure H_2_ annealing.^51^ It is recognized, however, that atmospheric pressure treatments afford layers that are gray rather than genuinely black and do not generate the room temperature, stable, isolated Ti^3+^ defect structure required for cocatalyst-free hydrogen evolution. Alternative reduction routes comprise the electrochemical (cathodic) polarization employed in the present work, which has been applied to enhance the photoelectrochemical response and the capacitance of TNT layers,^20,52,53^ and solution-based comproportionating reactions between TiO_2_ and metallic Ti, which blacken both nanotubes and nanoparticles under mild conditions.^54,55^

However, the literature lacks systematic and in-depth studies on the effects and impacts of the experimental variables employed in both the synthesis and self-doping processes of TiO_2_ films as well as on how these variables may influence the proportions of different crystalline phases of TiO_2_ and its electrochemical properties. Consequently, the novelty of the present study does not reside in the reduction of TNT layers itself, which is well established, but in the systematic and multivariate quantification of how the cathodic potential, the polarization time, and the calcination temperature jointly determine the extent of reduction and, consequently, the interfacial electrical response of the resulting electrodes. In this context, this study employs a factorial design to simultaneously investigate the effects of experimental variables such as the cathodic potential applied in the self-doping process, self-doping time, and calcination temperature on the phase composition and electrochemical properties of SD-TNT films. A full factorial design (FFD) and the response surface methodology (RSM) were applied to evaluate the effects of these parameters on the properties of the SD-TNT electrode and optimize the experimental conditions, respectively.^56^ The synthesized SD-TNT films were characterized by X-ray diffraction (XRD) with Rietveld refinement, Raman spectroscopy, field-emission gun scanning electron microscopy (FEG-SEM), electrochemical impedance spectroscopy (EIS), and cyclic voltammetry (CV). Diffuse reflectance spectroscopy (DRS) was also applied to investigate the optical and electronic properties of TNT films. Finally, from a technological standpoint, the results obtained in this work may contribute to the development of nanostructured TiO_2_ films with an enhanced optical and electronic efficiency.

## 2 Experimental Section

### 2.1 Chemicals and Materials

Titanium sheets (99.9% purity), ammonium fluoride (≥99.99% purity), glycerol (99.8% purity), and sodium sulfate (99.9% purity) were obtained from Sigma-Aldrich. Acetone (99.5% purity) and potassium chloride (≥99.0% purity) were obtained from Synth. Ruthenium dioxide sheets were obtained from De Nora. All the solutions were prepared with high-purity water using a Millipore Milli-Q system (resistivity >18 MΩ cm at 25 °C).

### 2.2 Synthesis and Experimental Optimization of SD-TNT Films

The TNT films were synthesized according to a methodology adapted from our previous reports.^7,8,41^ Initially, polished titanium sheets (1.0 × 0.5 cm) were sequentially ultrasonicated in acetone, isopropyl alcohol, and ultrapure water for cleaning. The TNT films were prepared by anodization of the cleaned titanium sheets at a constant potential (i.e., under potentiostatic conditions) of 30 V for 24 h in a glycerol solution containing 0.25 wt % NH_4_F and 10 vol % water. The target potential was attained by applying a linear potential ramp of 2.0 V min^–1^ using a power supply unit (MPS-3005, Minipa). A conventional two-electrode cell was used, employing titanium and ruthenium sheets (1.0 × 0.5 cm) as the anode and cathode, respectively. The obtained TNT films (applied as electrodes) were submitted to heat treatments to convert the amorphous TNT films into crystalline phases.

Subsequently, the SD-TNT films were obtained by cathodic polarization on a PalmSens 4 potentiostat/galvanostat, using a three-electrode cell, with a platinum sheet and Ag/AgCl (KCl 3.0 mol L^–1^) as auxiliary and reference electrodes, respectively. At this stage, a 0.10 mol L^–1^ Na_2_SO_4_ solution was used as an electrolyte. To systematically assess the influence of independent variables such as cathodic potential applied in the self-doping process, self-doping time, and the calcination temperature on the phase composition and electrochemical properties of SD-TNT films, as well as to optimize the experimental synthesis conditions, a full factorial design (FFD) combined with RSM was employed (Table 1).^8,56^ The experimental variables were investigated within the following ranges: cathodic potentials from −1.0 to −1.5 V (vs Ag/AgCl, KCl 3.0 mol L^–1^) for self-doping, 10 to 480 min of self-doping time, and 450 to 650 °C for calcination temperature. The independent variables were normalized and coded at levels −1, 0, and +1, corresponding to the low, center, and high levels, respectively.^56,61^ The cathodic current density (*j*_c_) corresponding to the electrochemical reduction of Ti^4+^ to Ti^3+^ was chosen as the observed response (i.e., dependent variable) and utilized as the response parameter in the experimental design. This response was correlated with the coded values (i.e., independent variables) using a general polynomial eq 1 ^56,61^

1 $$Y=\beta_{0}+\sum_{i=1}^{k}\beta_{i}x_{i}+\sum_{i=1}^{k}\sum_{i\neq j=1}^{k}\beta_{ij}x_{i}x_{j}+\sum_{i=1}^{k}\sum_{i\neq j=1}^{k}\sum_{i\neq l=1}^{k}\beta_{ijl}x_{i}x_{j}x_{l}+\varepsilon$$

where *Y* represents the observed response, β_0_ is a constant coefficient (intercept), β_*i*_ denotes the coefficient of the *i*-th effects, β_*ij*_ and β_*ijl*_ represent the coefficients for two and three-way interactions, respectively, *k* is the number of independent variables (three in our study), and ε is the random error.

**Table 1:** Coded Levels and Real Values for the Full Factorial Design (FFD) Used in the RSM Optimization of SD-TNT Synthesis^a^

run	coded levels			real values			response
	*X*_1_	*X*_2_	*X*_3_	*E*/V	time/min	*T*/°C	*j*_c_/mA cm^–2^
1	+1	+1	+1	–1.0	480	650	–0.003
2	+1	+1	–1	–1.0	480	450	–0.212
3	+1	–1	+1	–1.0	10	650	–0.002
4	–1	+1	+1	–1.5	480	650	–0.007
5	+1	–1	–1	–1.0	10	450	–0.153
6	–1	+1	–1	–1.5	480	450	–0.325
7	–1	–1	+1	–1.5	10	650	–0.005
8	–1	–1	–1	–1.5	10	450	–0.202
9	0	0	0	–1.25	225	550	–0.237
10	0	0	0	–1.25	225	550	–0.241
11	0	0	0	–1.25	225	550	–0.224

a The observed response was the cathodic current density (*j*_c_) for the electrochemical reduction of Ti^4+^ to Ti^3+^ in 0.10 mol L^– 1^ Na_2_SO_4_.

To evaluate and validate the developed model through analysis of variance (ANOVA), three replicates were performed at the midpoint of the independent variables (runs 9–11). The assays were randomly performed to minimize systematic errors. Finally, the responses were analyzed using the least-squares method and response surfaces were generated using StatSoft STATISTICA v.10 software to identify the optimal synthesis conditions for the SD-TNT films.

### 2.3 Characterization of TNT and SD-TNT Films

Morphologic characterizations of the synthesized TNT films were performed using high-resolution scanning electron microscopy (FEG-SEM) FEI MAGELLAN 400L coupled with energy-dispersive X-ray spectroscopy (EDS). Structural characterizations were performed using X-ray diffraction (XRD) on a PANalytical XPert. PRO MPD diffractometer at 40 kV and 30 mA, equipped with CuKα radiation, over a 2θ range of 5 to 80°. Morphological and structural characterizations were not conducted for the SD-TNT films, as self-doping does not significantly alter their morphology or crystallinity.

Optical and electronic properties of the synthesized films were investigated by diffuse reflectance spectroscopy (DRS) using a Shimadzu Spectrophotometer, model UV-2600, operating in the range of 200 to 800 nm. Based on the DRS results, the band gap values of the films were estimated by extrapolating the linear region to (α*hv*)^1/2^ of the Tauc plots, calculated from Kubelka–Munk’s function (2), according to our previous reports.^36,62,63^

2 $$\alpha hv=A{\left(hv-E_{g}\right)}^{1/2}$$

where α, *h*, ν, *A*, and *E*_g_ are the extinction coefficient, Planck’s constant (J s), frequency of the incident light (s^–1^), absorption constant, and gap energy (eV), respectively.

Structural and optical properties of the synthesized films were also investigated using Raman analysis with a WITec Confocal Raman Microscope System Alpha300+ (λ_max_ = 532 nm) and a ZEISS objective at 50× magnification. All spectra were obtained in a window range of 0 to 4000 cm^–1^, with a resolution of 2 cm^–1^, and applying 10 s of integration time and 30 scans.

The SD-TNT films (1.0 cm^2^) were electrochemically characterized by cyclic voltammetry (CV) and electrochemical impedance spectroscopy (EIS) on a PalmSens 4 potentiostat/galvanostat, using a conventional three-electrode cell, with Ag/AgCl (KCl 3.0 mol L^–1^) and a platinum mesh (2.0 cm^2^) as reference and auxiliary electrodes, respectively. A 0.10 mol L^–1^ Na_2_SO_4_ solution was employed as the supporting electrolyte. The cyclic voltammograms were performed in a potential range of −1.5 to 1.5 V (vs KCl 3.0 mol L^–1^) and at a scan rate of 75 mV s^–1^. The impedance spectra were obtained at open-circuit potential (OCP) of −0.199 V (vs Ag/AgCl, KCl 3.0 mol L^–1^) and in the frequency range of 10 μHz to 10 kHz.

## 3 Results and Discussion

### 3.1 Characterization of TNT and SD-TNT Films

Figure 1a–c presents the top-view FEG-SEM images of the TNT films calcined at 450, 500, and 650 °C, respectively. In all cases, self-organized nanotube arrays were obtained, with inner diameters of 89.02 ± 4.83 nm, wall thicknesses of 24.56 ± 3.01 nm, and a layer thickness (i.e., tube length) of approximately 1.06 ± 0.14 μm, the latter determined from the cross-sectional image as shown in the inset, Figure 1a. These dimensions are consistent with those expected for anodization at 30 V in glycerol-based electrolytes and are relevant to the discussion that follows because the tube length and the inner diameter define the developed surface area and the charge-transport length of the layers, and hence the interfacial response analyzed in Section 3.3.^57–62^ Comparison of the three images shows that the calcination temperature, within the range investigated, does not modify the morphology, so that the differences observed in the subsequent characterizations arise from changes in crystallinity rather than in geometry.

**Figure 1:**
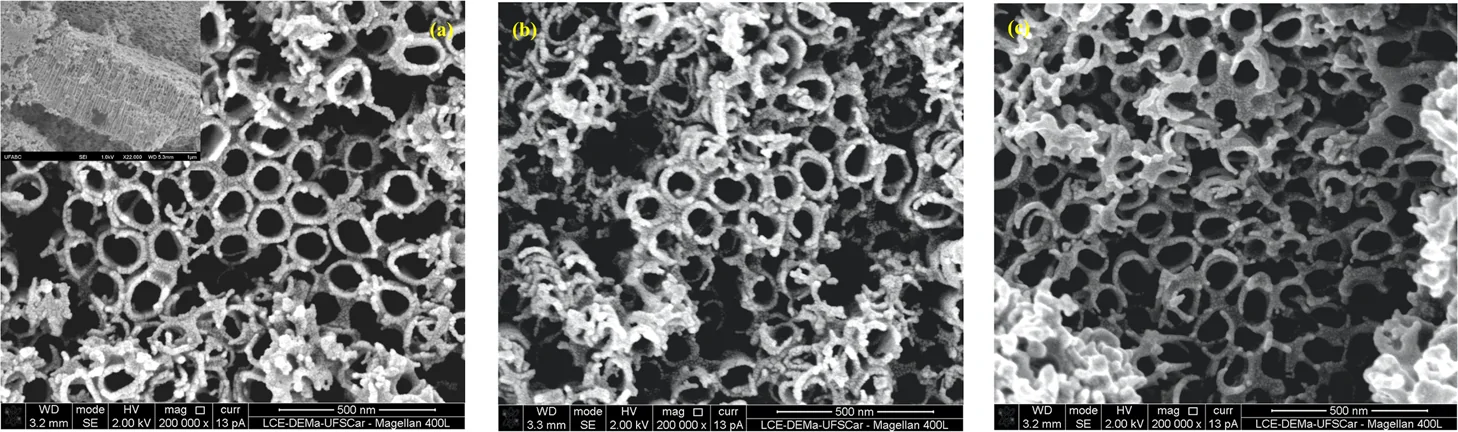
Top view FEG-SEM images of the TNT films formed in the substrate of titanium calcinated at different temperatures (a) 450, (b) 550, and (c) 650 °C.

The layers are nevertheless not defect-free, presenting local irregularities; partially collapsed tube mouths and residual debris between the tubes are apparent in all images and are characteristic of the long anodizations performed here in a viscous glycerol-based electrolyte at room temperature without a subsequent cleaning step, which are associated with the degree of ordering that is thus lower than that attainable in optimized ethylene-glycol-based electrolytes. Furthermore, the material used to obtain the images—carbon tape—may also have played a role in this regard. Superimposed on these intrinsic features, the granular contrast observed at higher magnifications is ascribed to the carbon clusters formed during the metallization required for imaging and should therefore not be interpreted as a property of the oxide. Neither type of imperfection compromises the conclusions drawn below, since all electrochemical comparisons are made between TNT and SD-TNT electrodes prepared from the same batch, whose morphology is unaffected by the calcination temperature and highly ordered.

The influence of calcination temperature on the proportion of crystalline phases (i.e., anatase and rutile) was further investigated through XRD with Rietveld refinement and Raman spectroscopy. Figure 2 shows the X-ray diffractograms for the synthesized TNT films without heat treatment and calcined at 450, 550, and 650 °C for 1 h. As can be seen in the diffractograms, no crystalline phases were detected in the TNT film without heat treatment (as expected), while the calcination temperature promoted the formation of anatase and rutile phases, with their relative proportions significantly influenced by applied temperature. The diffractograms for the calcined TNT films showed peaks indexed to anatase planes (101, 004, 202, 211, and 215) and rutile planes (110, 101, 201, and 301) (JCPDS cards 21-1272 and 21-1276, respectively). As expected, the TNT films calcined at 450 °C exhibited only the anatase phase. Raising the calcination temperature beyond this temperature led to a gradual decay in the proportion of the anatase phase, accompanied by the emergence of the rutile phase at 550 °C and its further growth at 650 °C (see planes 110, 201, and 301). In addition, it should be noted that indexed peaks corresponding to titanium planes (100, 112, 102, 204, and 220) for all films (i.e., without heat treatment and calcined at 450, 550, and 650 °C). The presence of Ti planes is due to the relatively small thickness of the TNT films (approximately 1.0 μm), which allows the X-ray beam to penetrate through the TNT film and reach the titanium substrate.

**Figure 2:**
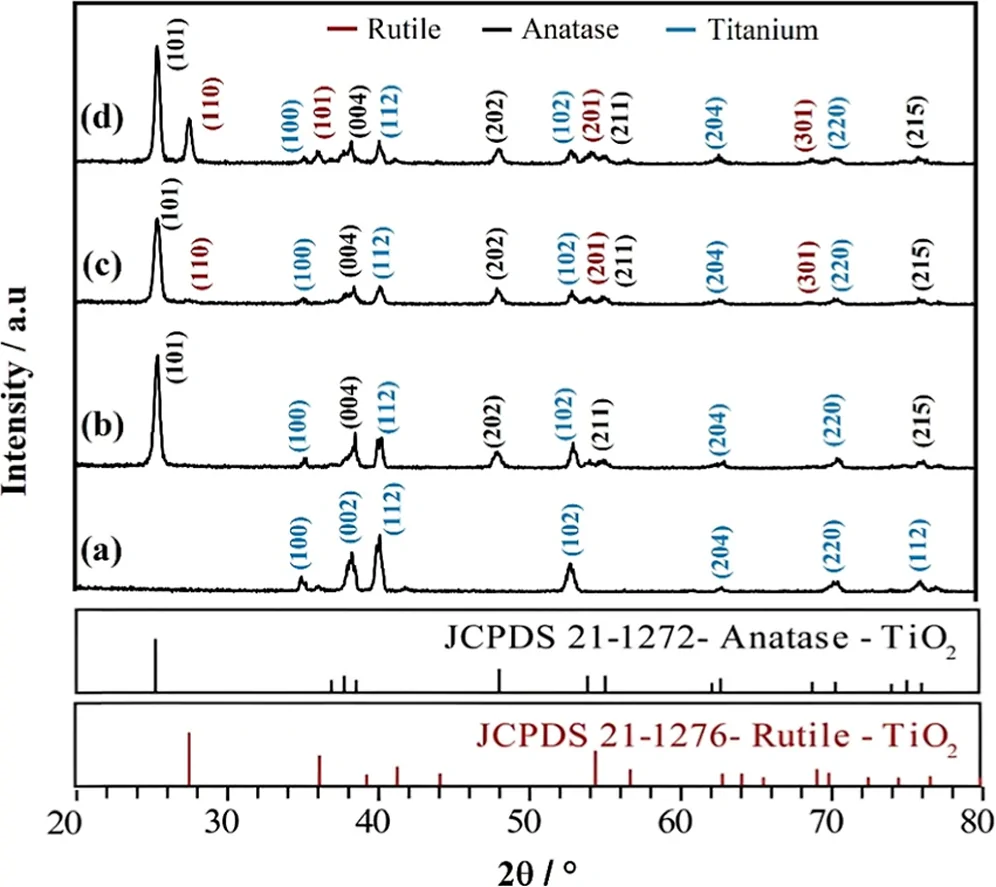
XRD spectra of (a) pristine titanium and TNT films calcined for 60 min at (b) 450 °C, (c) 550 °C, and (d) 650 °C.

Rietveld refinements were applied to the diffractograms (see Figure 2) at different temperatures (i.e., 450, 550, and 650 °C) to estimate the relative proportions of the crystalline phases. As can be seen in Figure S3, the results showed the trend observed in the diffractograms illustrated in Figure S3, in which no crystalline phase was observed for the TNT film before heat treatment. However, for the TNT film subjected to calcination at 450 °C, only the anatase crystalline phase was found, as expected, and previously shown in Figure 2, corroborating findings reported in the literature for nanoparticles.^64^ The anatase content phase decreased from 51% for the heat treatment at 450 °C to 48% at 550 °C and 15% at 650 °C, accompanied by the onset of the rutile phase at 550 °C (planes 110 and 101 in Figure 2) and its pronounced growth at 650 °C. The rutile phase contents were estimated to be 15% and 38% for the TNT films heat-treated at 550 and 650 °C, respectively. These results confirm that higher treatment temperatures favor the formation of the rutile phase at the detriment of the anatase one. Furthermore, for the film treated at 550 °C, the phase distributions (disregarding the percentage ascribed to the substrate) were 76% and 24% for anatase and rutile, respectively, a ratio considered optimal for photocatalytic applications and commercially available (TiO_2_ P25).^34,65^ The self-doping was not detected in XRD analysis. Rietveld refinement parameters and the crystallite sizes of the phases (anatase and rutile) obtained as a function of calcination treatment at 450, 550, and 650 °C using the Size-Strain Plot (SSP) and Scherrer methods are illustrated in Figures S1 and S2, respectively.

The Tauc plots obtained from the DRS analysis of TNT and SD-TNT films calcined at 450, 550, and 650 °C are presented in Figure S4a,b, respectively. By extrapolating the linear region for all curves to the point where (α*h*ν)^1/2^ equals zero, it was possible to estimate the apparent band gap values for all TNT films, crucial to evaluate the optical response, and, consequently, the ability of these materials to generate electron/hole pairs when exposed to radiation sources. The band gap values of the pristine and SD-TNT films were found to be dependent on the calcination temperature. For TNT films calcined at 450, 550, and 650 °C, the values were 3.23, 3.21, and 3.32 eV, respectively. After 480 min of cathodic polarization (i.e., self-doping), the band gap values of the films decreased to 3.18, 3.09, and 3.27 eV, ascribed to the introduction of Ti^3+^ dopant states and modifications of the surface properties.^66,67^ Despite the decrease in band gap values caused by self-doping, the TNT and SD-TNT films showed similar tendency, with the lowest band gap values recorded for films heat-treated at 550 °C (medium value), while higher values were found for the films calcined at 450 and 650 °C (see Figure S4). As shown in Figure S3, the anatase content decreased and the rutile content increased almost linearly with increasing calcination temperature. By comparing both, we inferred that the better optical response was achieved for the SD-TNT film with an anatase/rutile ratio of 76/24 (in %), determined by Rietveld refinement (see Figure S3).

Moreover, the band gap values can be influenced by the spatial distribution of the atoms within the TiO_2_ crystalline structures. For the rutile phase, the closer interaction between occupied and empty orbitals 2p and 3d of oxygen and titanium, respectively, leads to a band gap of 3.00 eV. This lower value, compared to the band gap of the anatase phase (3.20 eV), arises from the more compact and symmetric structure of the rutile phase, which results in a reduced conduction band splitting and, consequently, a smaller band gap value for the crystalline rutile phase.^33,68,69^ According to Mi and Weng,^33^ five possible band alignments can occur between anatase and rutile phases of TiO_2_, depending not only on their relative phase proportions but also on the particle’s sizes. Each of these five alignments reflects different relationships between the conduction and valence band energies of the rutile and anatase phases, which can significantly influence the electronic and optical properties of TiO_2_-based materials. These band alignments established in the heterojunction region give rise to intermediate energy levels, commonly associated with oxygen vacancies, structural defects, and/or lattice distortions. As a result, charge redistribution occurs at the anatase/rutile interface, leading to the formation of energy states within the band gap, and the interfacial electron transfer between the phases modifies the local Fermi level.^70^ The practical consequence is the reduction of the apparent band gap of the semiconductor, even though the intrinsic band gap of the individual phases (anatase and rutile) remains unaltered.

The band alignments endorse the apparent band gap of 3.09 eV obtained for the SD-TNT film calcined at 550 °C, an intermediate value between those of the anatase (3.20 eV) and rutile (3.00 eV) phases. Furthermore, as previously mentioned, crystallite sizes may also influence the band alignments in the heterojunction regions.^33,71^ This could explain the higher apparent band gap values observed for both TNT (3.32 eV) and SD-TNT (3.27 eV) films calcined at 650 °C, even with an increase in the proportion of rutile phase (71%). As shown in Table S2 (Supporting Information), the estimated crystallite sizes of the anatase and rutile phases observed were larger for the films calcined at 650 °C. For the anatase phase, the estimated sizes by SSP and Scherrer methods increased from 218.60 and 212.06 Å, respectively, at 550 °C to 237.37 and 231.36 Å when calcined at 650 °C. A similar trend was observed for the rutile phase, where the estimated crystallite sizes by SSP and Scherrer methods increased from 93.35 and 88.38 Å, respectively, calcined at 550 °C to 267.81 and 270.77 Å for the films calcined at 650 °C. These results indicate that the reduction in the apparent band gap is influenced by both the anatase/rutile ratio and crystallite size, which synergistically promote a more effective band alignment within the heterojunction region.

Raman spectroscopy was applied for structural characterization of the TNT and SD-TNT films heat-treated at temperatures of 450, 550, and 650 °C. In addition, confocal Raman imaging was employed to qualitatively evaluate the spatial distribution of anatase and rutile phases. Two distinct regions, identified by contrasting colors (darker and lighter), were analyzed as a function of their composition (see Figure S1 in the Supporting Information). Figure 3a,b show the Raman spectra of the TNT and SD-TNT films calcined at different temperatures. For the films thermally treated at 650 °C, spectra from two distinct regions (designated as A and B), corresponding to the darker and lighter regions, are also presented.

**Figure 3:**
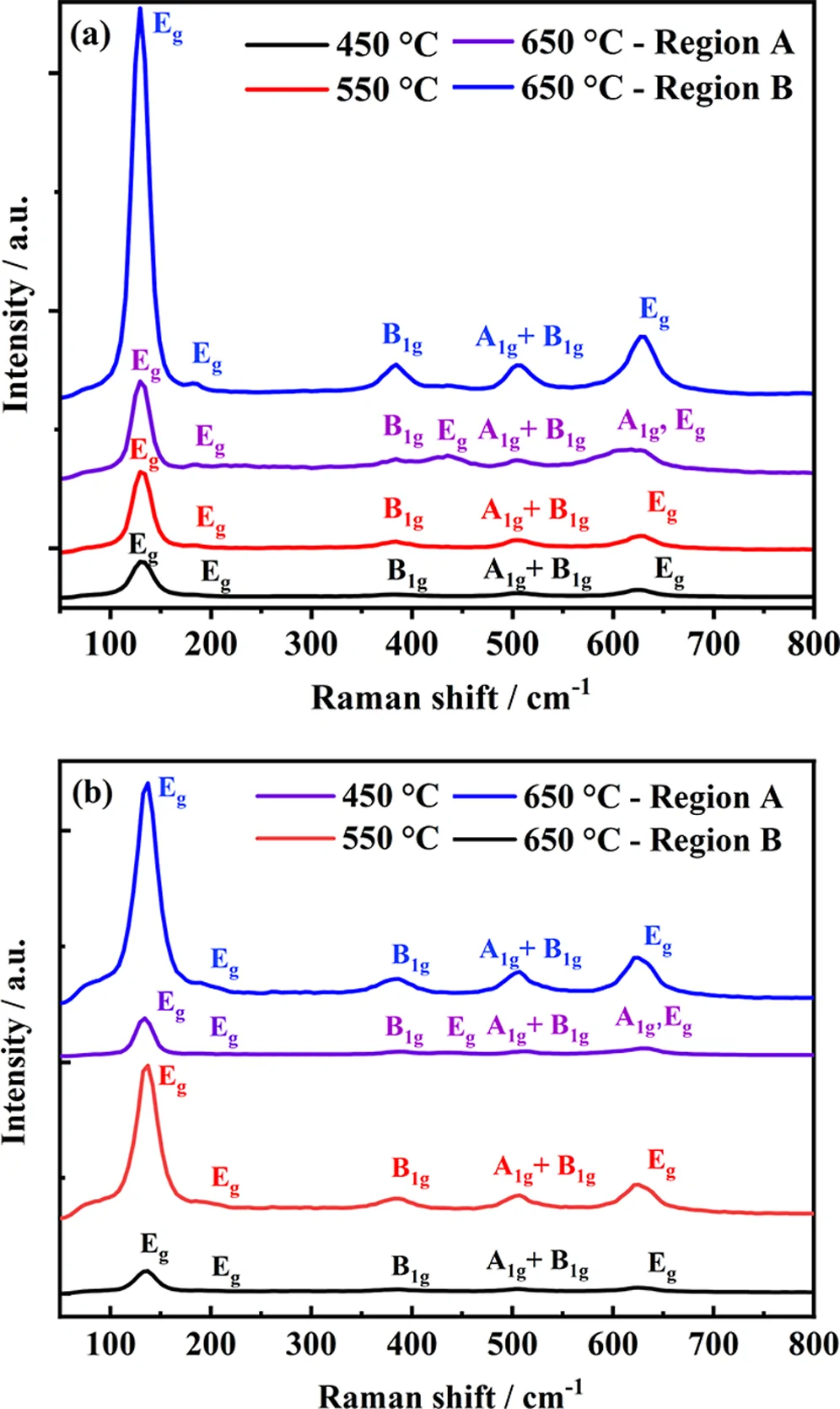
(a) Raman spectra of TNT films calcined for 60 min at different temperatures, with the corresponding vibrational modes indexed. (b) Raman spectra of SD-TNT films calcined for 60 min at different temperatures, with the corresponding vibrational modes indexed. (λ = 532 nm). All SD-TNT films were self-doped for 450 min under −1.5 V.

As shown in Figure 3a,b, the TNT and SD-TNT layers calcined at 450 and 550 °C display the five Raman-active modes of anatase at 132 (*E*_g_), 185 (*E*_g_), 383 (*B*_1g_), 506 (*A*_1g_ + *B*_1g_), and 626 cm^–1^, whose assignment is well established for anodic TNT layers and is therefore not discussed in detail here.^72,73^ For the layers calcined at 650 °C, the spectra collected in region A additionally exhibit the rutile modes at 432 (*E*_g_) and 617 cm^–1^ (*A*_1g_ partially overlapping *E*_g_), whereas region B remains essentially anatase-like, confirming the spatial heterogeneity of the anatase-to-rutile conversion.^74^ The only spectral feature that differs systematically between TNT and SD-TNT is the blue-shift of the *E*_g_ mode after cathodic polarization, which is examined below by deconvolution.

The deconvolutions of the anatase *E*_g_ mode (∼135 cm^–1^ are compared in Figure 4a–d, for TNT; and in Figure 5a–d, for SD-TNT). Since this mode responds both to the anatase content and to the presence of oxygen vacancies, the ratio between its stoichiometric and vacancy-related components was used as an indicator of the extent of reduction.^75–77^ For the TNT layers calcined at 450 and 550 °C, the stoichiometric/vacancy intensity ratios were 2.0:1.0 and 3.0:1.0, respectively, and after cathodic polarization they were inverted to 1.0:1.5 and 1.0:1.7, confirming that the self-doping process increases the density of oxygen vacancies and, consequently, of Ti^3+^ centers. For the layers calcined at 650 °C, the analysis is no longer quantitative because the B_1g_ mode of rutile appears in the vicinity of the anatase E_g_ band at 132 cm^–1^ and the deconvolution does not return a coherent stoichiometric/vacancy ratio. However, even in this case, the vacancy-related component increases after polarization. The overall increase of the Raman scattering intensity with the calcination temperature, observed for region B, simply reflects the well-documented improvement of crystallinity with thermal treatment.^46,77^

**Figure 4:**
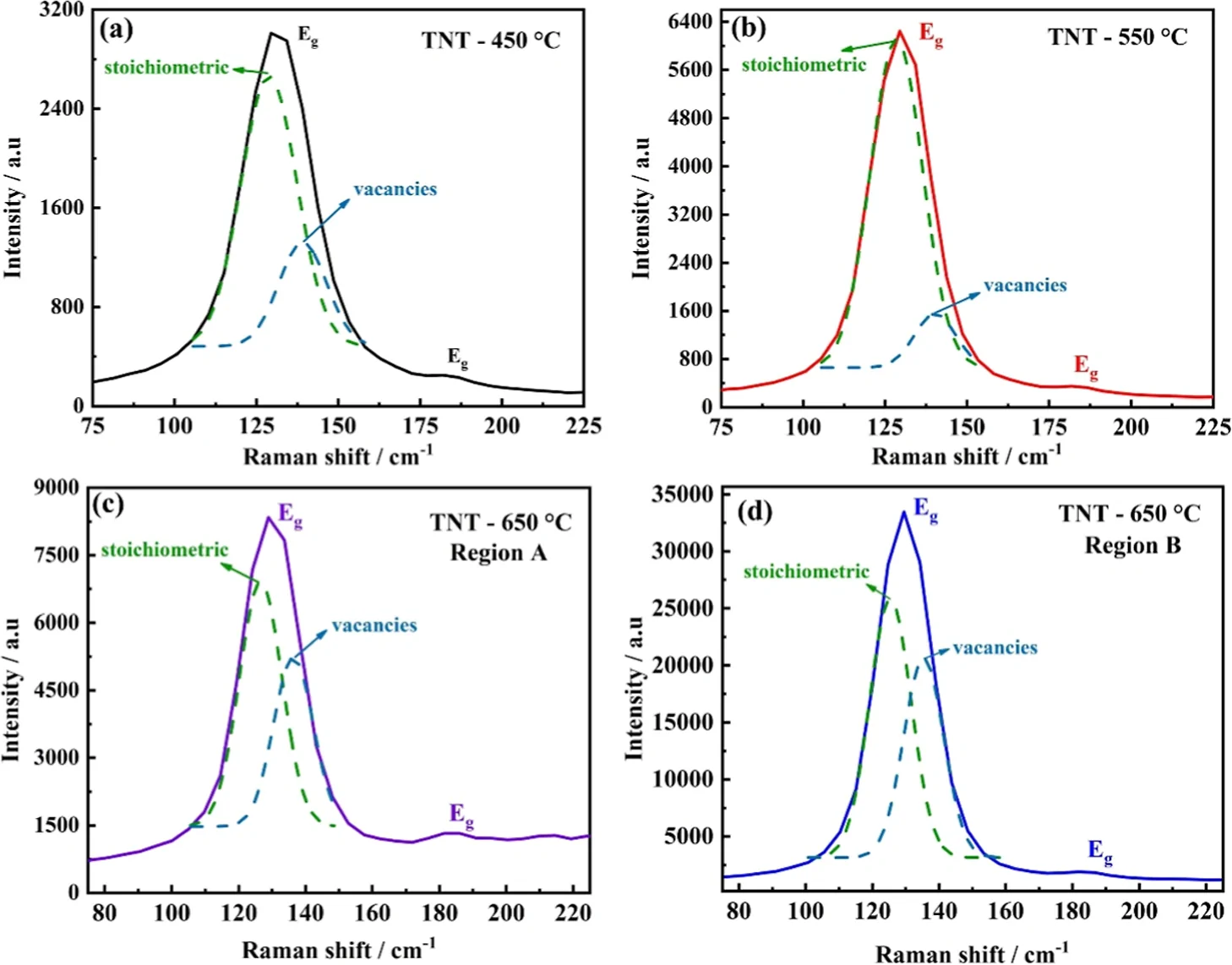
(a–d) Raman spectra of TNT films calcined for 60 min at 450, 550, and 650 °C, respectively, along with the deconvolution of the active *E*_g_ mode at 132 cm^–1^.

**Figure 5:**
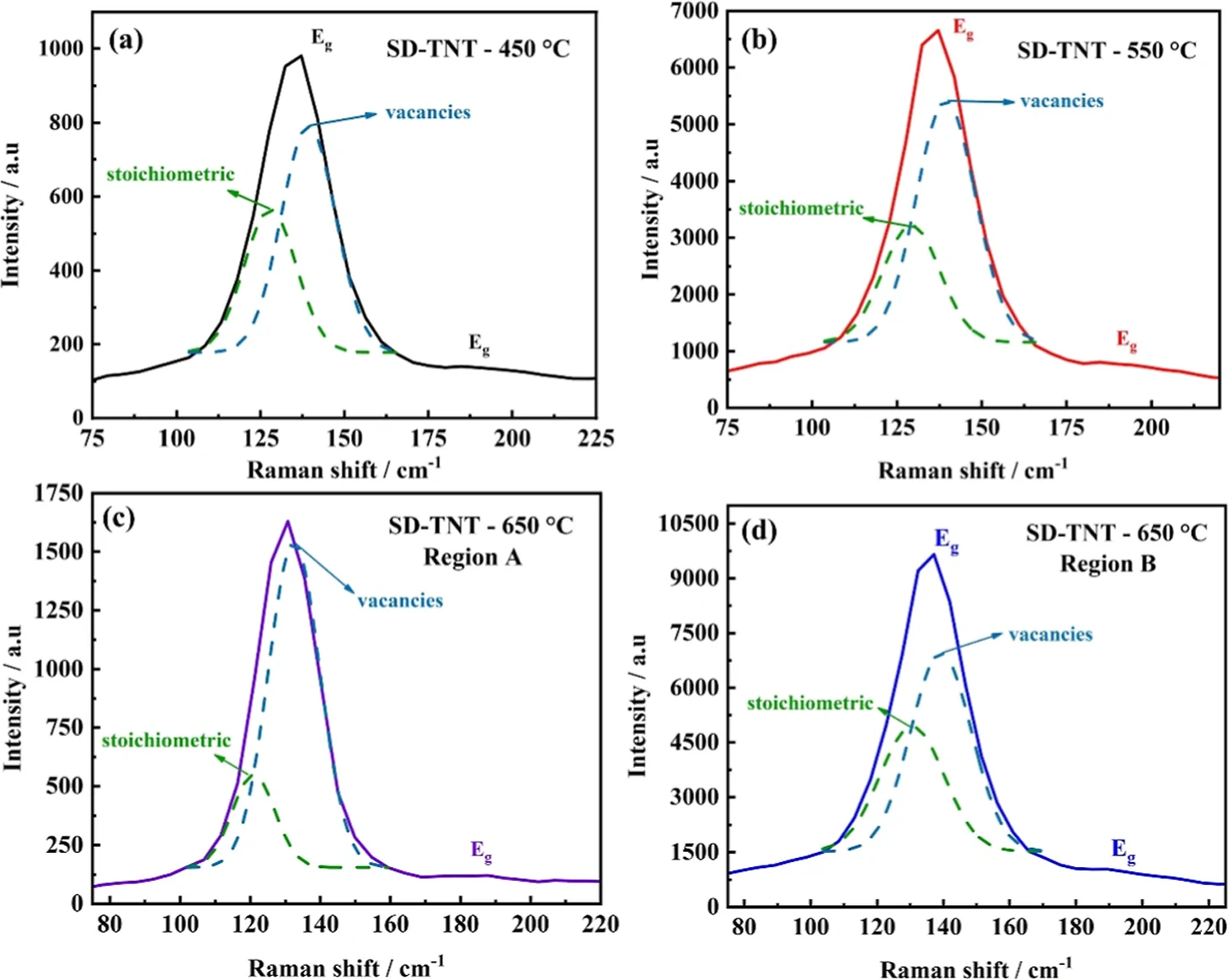
(a–d) Raman spectra of SD-TNT films calcined for 60 min at 450, 550, and 650 °C, with the corresponding deconvolution of the *E*_g_ mode at 132 cm^–1^. All SD-TNT films were self-doped for 450 min under −1.5 V.

To better assess rutile phase formation, regions A and B (as previously mentioned) were analyzed for films calcined at 650 °C. Under this heat-treatment condition, two additional bands at 432 and 617 cm^–1^ attributed to the *E*_g_ and *A*_1g_ (partially overlapping with *E*_g_) vibrational modes, respectively, are associated with the rutile phase and can be used to evaluate its formation. Then, a qualitative estimation of rutile phase formation and the anatase/rutile ratio can be performed by monitoring the deconvoluted band located at 617 cm^–1^ (*E*_g_) using Gaussian functions, as illustrated at Figure S2 in the Supporting Information. Since the *A*_1g_ mode is associated with the rutile phase, whereas the *E*_g_ is associated with the anatase phase, it is reasonable to presume the presence of the rutile phase in region A. However, the Rietveld method provides a more reliable estimate of the anatase/rutile phase ratios. Furthermore, the more intense band associated with the rutile phase in region A explains why the Raman scattering intensities of both TNT and SD-TNT in this region did not follow the trend observed for region B, i.e., an increase in Raman scattering intensities with rising calcination temperature.

Given that self-doping of TNT films involves the reduction of Ti^4+^ to Ti^3+^, thereby promoting the formation of oxygen vacancies and consequently enhancing their electrochemical conductivity, cyclic voltammograms were obtained for films subjected to different self-doping durations to better understand the effects of the self-doping process. Figure 6a illustrates the cyclic voltammograms of SD-TNT films self-doped at −1.5 V (vs Ag/AgCl, KCl 3.0 mol L^–1^) in the range of 0 to 480 min and calcined at 450 °C. As can be seen, all voltammograms exhibited typical profiles of the anatase phase, with two characteristic peaks in the −1.30 to −1.17 V and −0.80 to 0.65 V ranges, attributed to the oxidation of Ti^3+^ to Ti^4+^ and the reduction of Ti^4+^ to Ti^3+^, respectively. Additionally, the oxidation peaks shifted toward more cathodic overpotentials with increasing self-doping duration, whereas the reduction peaks shifted toward lower overpotentials as a function of the self-doping time. These simultaneously shifts indicate that the self-doping has promoted an increase in the Ti^3+^ concentration and, consequently, enhanced the conductivity and lowering the cathodic overpotential necessary for Ti^4+^ reduction.^78^ Finally, it is observed that the cathodic current attributed to the Ti^4+^ reduction increased with self-doping duration (see Figure 5b), confirming that this variable is an important parameter to optimization of SD-TNT synthesis.

**Figure 6:**
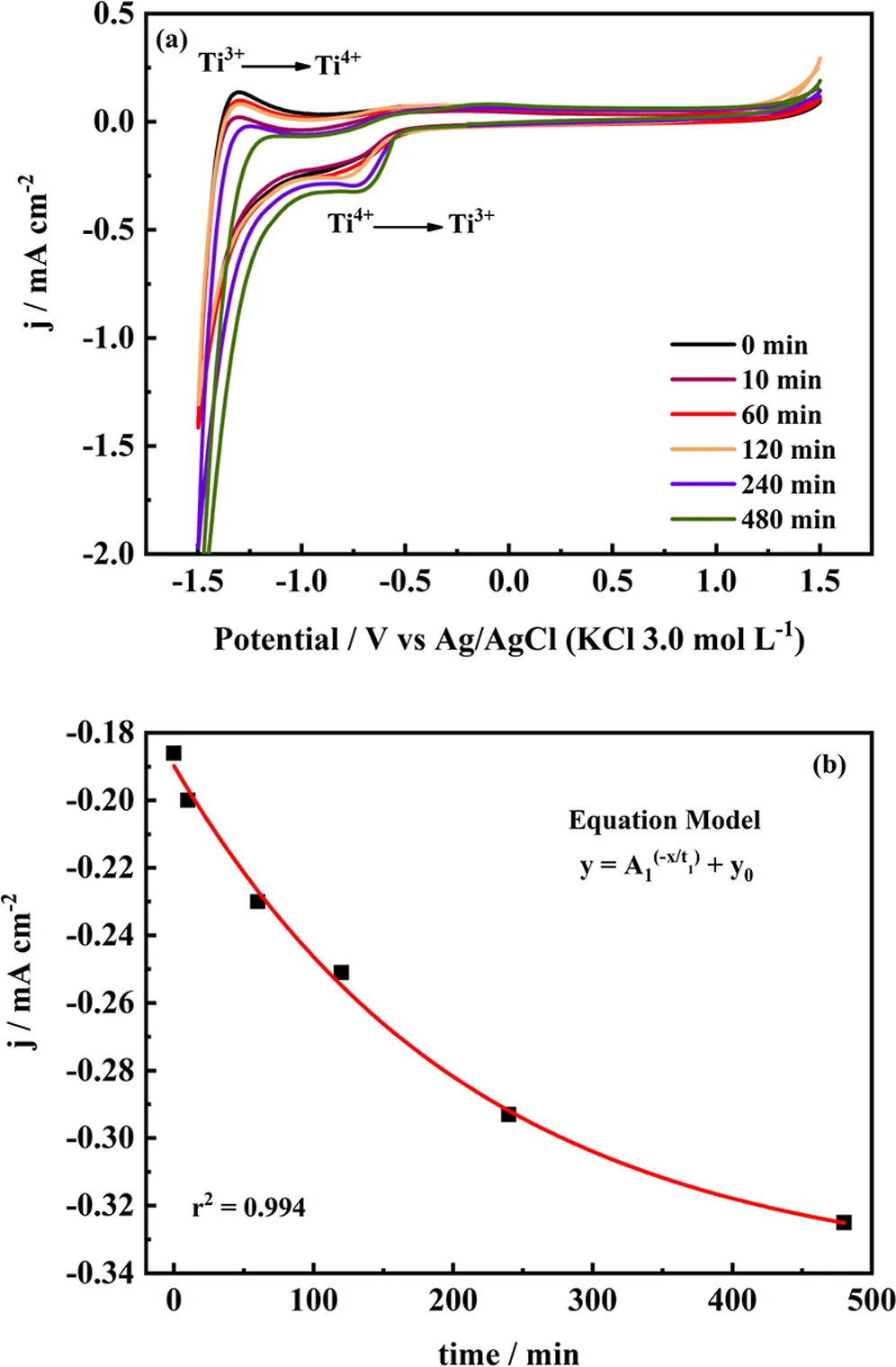
(a) Cyclic voltammograms of the SD-TNT films calcined at 450 °C and self-doped at −1.5 V, for different doping times. (b) Relationship between doping time and current density. Supporting electrolyte of Na_2_SO_4_ 0.10 mol L^–1^, cathodic scan from 1.5 to −1.5 V at a scan rate of 75 mV s^–1^.

### 3.2 Optimization of Experimental SD-TNT Synthesis

Table 1 shows the observed response (*j*_c_) as a function of the experimental variables: the cathodic potential applied during the self-doping process, the self-doping time, and the calcination temperature. The response was evaluated using a multiple linear regression based on the least-squares method and using data from runs 1–11. Equation 3 was generated using a first-order model of the observed response *j*_c_, incorporating only the statistically significant terms at 95% confidence level

3 $$jc=-0.146+0.021x_{1}-0.023x_{2}+0.109x_{3}-0.019{x_{1}x}_{3}+0.022x_{2}x_{3}$$

were *x*_1_, *x*_2_, and *x*_3_ are the coded variables corresponding to the cathodic potential applied in the self-doping process, self-doping time, and the calcination temperature, respectively.

The mathematical model demonstrates a good linear correlation between the observed and predicted *j*_c_ values (considering a multivariate model), with a square regression coefficient (*R* ^2^) of 0.780. Figure S5a confirms this correlation, showing strong concordance between the predicted (calculated from eq 3) and experimental values for *j*_c_. Figure S5b depicts the residual-predicted plot, which exhibits a random distribution around zero deviation, indicating the absence of systematic errors and consistency with the normal distribution expected from the *F*-test.^60^ Based on these considerations, the effects of the experimental variables on the performance of the SD-TNT films were evaluated using the cathodic current density (*j*_c_) associated with the electrochemical reduction of Ti^4+^ to Ti^3+^, using the response surfaces generated from eq 3 and illustrated in Figure 7a,b.

**Figure 7:**
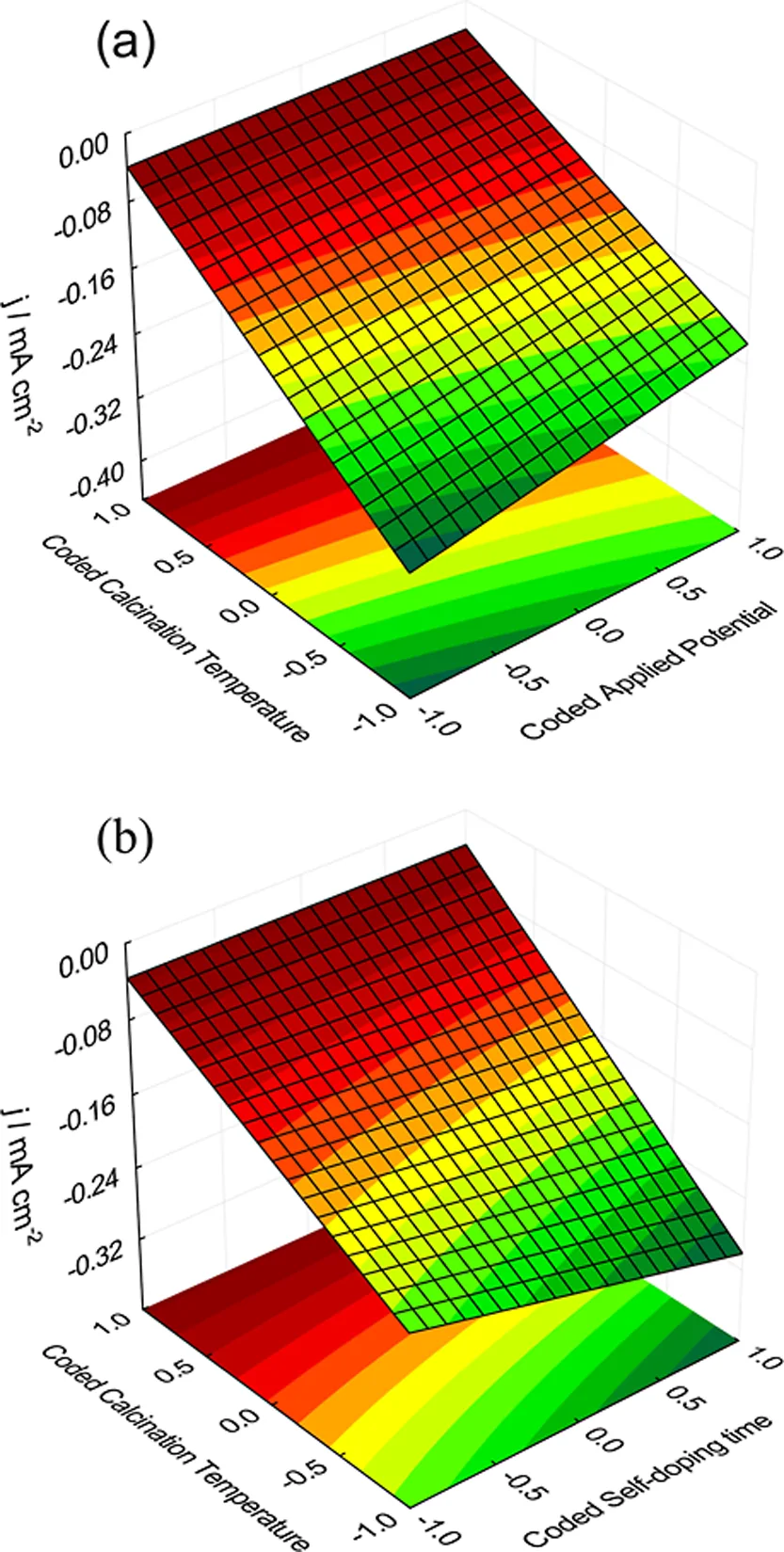
Response surface plots generated from a full factorial design (FFD) using eq 3 showing *j*_c_ as a function of (a) potential versus calcination temperature and (b) self-doping time versus calcination temperature.

Figure 7a,b present response surfaces derived from the first-order model related to *j*_c_ as a function of cathodic potential, calcination temperature, self-doping time, and calcination temperature, respectively. As can be seen, Figure 7a shows a gradual increase in *j*_c_ as the cathodic overpotential increases from −1.0 to −1.5 V for the films submitted to thermal treatment at 450 °C. However, the *j*_c_ values progressively dropped with raising the calcination temperature from 450 to 650 °C. This behavior can be related to a gradual decrease in the proportion of the anatase phase and the emergence of the rutile phase at 550 °C, with further growth at 650 °C, as previously discussed. In other words, an increase in rutile content can modify the band alignment, favoring the electron/lacune pair recombination, thereby reducing the conductivity and, consequently, *j*_c_.

The response surface shown in Figure 7b reveals a similar negative effect of calcination temperature on *j*_c_, with higher cathodic current densities achieved for films calcined at 450 °C. As expected, Figure 6b further indicates that *j*_c_ increases with self-doping time at the lower calcination temperature (i.e., at 450 °C), attributed to more effective self-doping, leading to a greater formation of Ti^3+^ sites and anatase/oxygen-vacancy defects, thereby enhancing the electrochemical conductivity of the SD-TNT films.^79^ In contrast, for films calcined at higher temperatures, self-doping time has no statistically significant effect on *j*_c_. This phenomenon is associated with increased rutile phase content, which modifies the band alignment, thereby promoting electron/hole recombination and reducing the electrochemical response (i.e., *j*_c_).

Based on the generated response surfaces, the optimum conditions for SD-TNT synthesis were established, considering the enhanced optical and electronic performance. Within the investigated ranges, the optimal parameters correspond to −1.5 V during 480 min to films calcined at 450 °C.

### 3.3 Electrochemical Characterization of SD-TNT under Optimized Conditions

The optimal synthesis conditions for SD-TNT (within the range of the studied variables) were determined by RSM, based on the response (*j*_c_) as a function of the independent variables cathodic potential applied in the self-doping process, self-doping time, and the calcination temperature. The optimum experimental conditions for the SD-TNT synthesis were found to be −1.5 V applied during 480 min and calcined at 450 °C. To gain deeper insights into the processes governing electrode performance, such as charge-transfer resistance, recombination, mass transport, and capacitive effects, EIS analyses were performed on the TNT electrode and on the SD-TNT electrode prepared under optimized conditions.

Figure 8a,b present the corresponding comparative Nyquist and Bode plots, respectively. The comparative Nyquist plots (Figure 8a) for the TNT and SD-TNT films clearly show that self-doping substantially reduced the charge-transfer resistance (*R*_ct_), decreasing from 831.9 Ω for the TNT electrode to 92.35 Ω for the SD-TNT electrode under optimized conditions. This reduction in *R*_ct_ is attributed to the formation of Ti^3+^ sites and oxygen-vacancy defects, which induce a lattice distortion and thereby enhance the electrochemical conductivity and facilitate electron transport across the SD-TNT interface.

**Figure 8:**
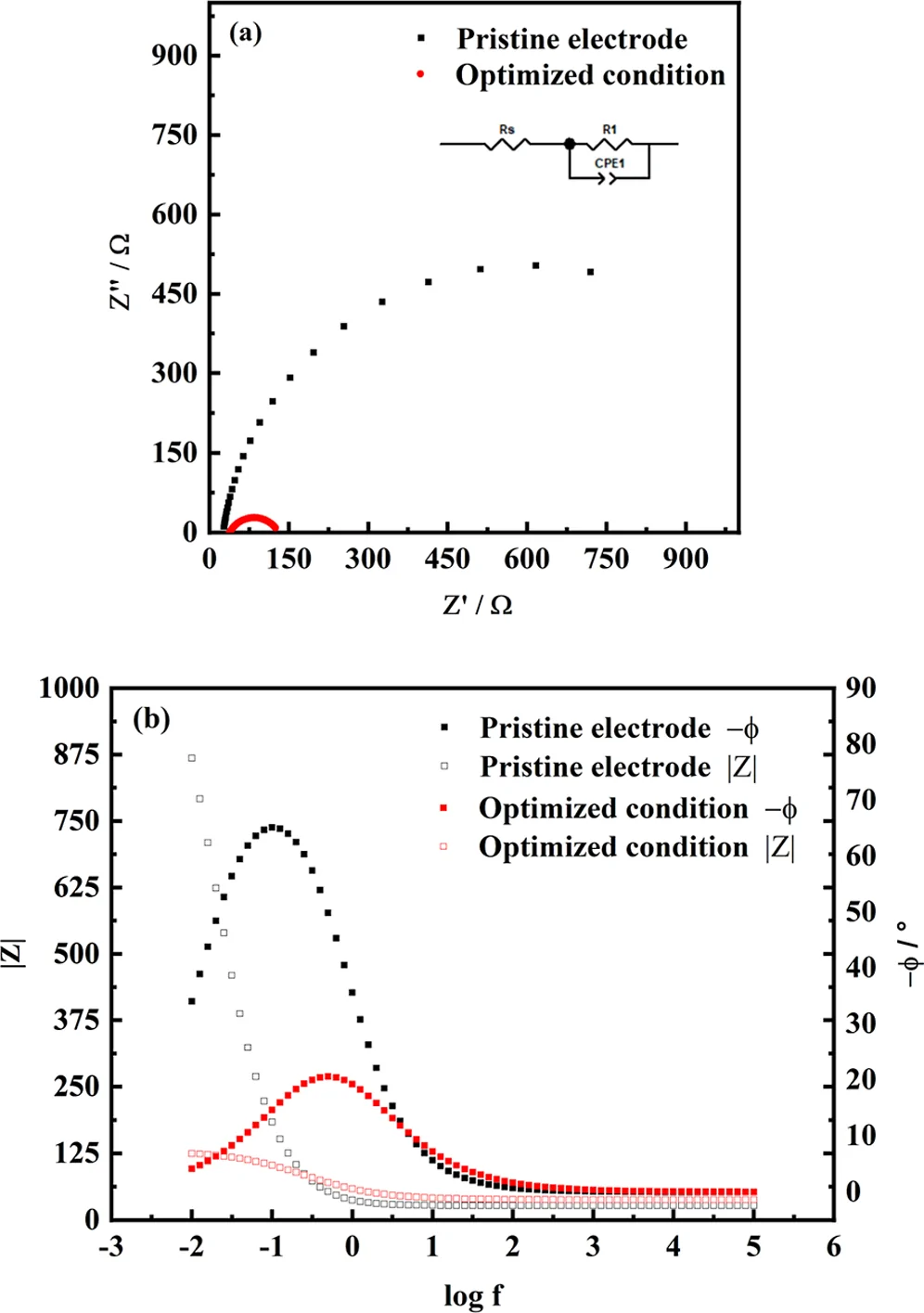
(a) Nyquist and (b) Bode plots for the pristine TNT film (calcined at 450 °C) and the SD-TNT film (obtained under optimized conditions). Measures were performed in Na_2_SO_4_ 0.1 mol L^–1^, and at *E*_OCP_ = −0.199 V over a frequency range from 100 kHz to 10 μHz.

According to the literature, the equivalent circuit that best describes the interfacial electrochemical processes in the SD-TNT electrode is the Randles circuit without the Warburg impedance element.^8,41^ Based on this model, the capacitance value was estimated by converting the constant phase element (*Q*) into an equivalent capacitance (C). The fitted EIS parameters are summarized in Table 2, indicating that self-doping under optimized conditions leads to a decrease in both capacitance and α compared with the TNT electrode. These decays in *C* and α values are ascribed to the reduction of surface Ti^4+^ species to Ti^3+^, which is associated with the reduction of capacitive behavior of the SD-TNT electrode compared with the pristine one.

**Table 2:** EIS Parameters Obtained from the Equivalent Circuit Models Used for TNT and SD–TNT Films at *E*_OCP_ = −0.199 V (vs Ag/AgCl 3.0 mol L^–1^), Measured Over a Frequency Range of 10 kHz to 10 mHz in 0.10 mol L^–1^ Na_2_SO_4_

	*R*_s_/Ω	*Q*/mF s^(1−α)^	α	*R*_ct_/Ω	C/μF	χ^2^/10^–3^
TNT	24.00	9.08	0.897	831.9	9079	1.3248
SD-TNT	38.00	7.37	0.692	92.35	8417	7.4679

The Bode diagrams (see Figure 8b) show the maximum phase angles decreasing for the SD-TNT electrode under optimized conditions compared to the TNT electrode, indicating a less pronounced capacitive behavior. This finding is consistent with expected results and corroborates capacitance values obtained from the model. Further, this model allowed an evaluation of the time constant, which was found to be lower for the SD-TNT electrode than for the TNT one, suggesting faster interfacial kinetics under optimized conditions.^80–82^ Nevertheless, the broader peak width obtained for the SD-TNT electrode suggests a wider distribution of time constants, implying that the interfacial process is more uniformly distributed in the interfacial region.^80,83^ Because the impedance response of a tubular electrode is intrinsically distributed along the tube axis, the fitted parameters were also examined in the light of the layer dimensions. Taking the tube length of 1.06 ± 0.14 μm, inner diameter of 89.02 ± 4.83 nm and 24.56 ± 3.01 nm, reported in Section 3.1, the geometric area of 1.0 cm^2^ corresponds to a developed (internal) area roughly one to 2 orders of magnitude larger, so that the area-normalized charge-transfer resistance and capacitance are correspondingly smaller than the values listed in Table 2. Two consequences follow. First, the decrease of *R*_ct_ upon cathodic polarization cannot be ascribed to a change in the developed area, which remains unaltered because the reduction does not modify the morphology. It therefore reflects a genuine increase in the density of Ti^3+^ centers and oxygen vacancies acting as electron donors, in agreement with the drop of the solid-state resistance reported for electrochemically self-doped TNT layers.^47^ Second, the lower CPE exponent (α) and the broader phase-angle peak obtained for the SD-TNT electrode are consistent with a reduction front that propagates from the tube mouth toward the tube bottom, generating a distribution of local time constants along the tube axis, in line with the distributed photoelectrochemical response described for self-organized TNT layers of different lengths.^57^ This interpretation also explains why the polarization time is statistically significant only for the layers calcined at 450 °C. In these anatase layers the reduction front can advance along the whole tube length, whereas the rutile formed at higher temperatures restricts its propagation.

## 4 Conclusions

FEG-SEM and XRD confirmed that the cathodic polarization does not alter the morphology or the crystalline structure of the layers, which consisted of self-organized nanotubes with inner diameters of 89.02 ± 4.83 nm, thickness of 24.56 ± 3.01 nm, and a tube length of 1.06 ± 0.14 μm. In agreement with the established behavior of anodic TNT layers, the calcination temperature governed the anatase-to-rutile conversion, and Rietveld refinement indicated an anatase/rutile percentage ratio of 76/24 for the electrode with the best optical response (due to a smaller band gap, as estimated by the Tauc plots, obtained from the DRS spectra). The Raman deconvoluted spectra allowed the quantification of the effect of the cathodic polarization on the anatase E_g_ mode: the stoichiometric/oxygen-vacancy intensity ratios of 2.0:1.0 and 3.0:1.0, obtained for the TNT layers calcined at 450 and 550 °C, were inverted to 1.0:1.5 and 1.0:1.7 after self-doping (i.e., for SD-TNT). However, for the films calcined at 650 °C, the overlap of *E*_g_ and the *B*_1g_ modes, this last one labeled for the rutile phase, prevents a coherent quantification.

Additionally, this study also reported a multivariate optimization of the experimental conditions for SD-TNT synthesis using a full factorial design (FFD) combined with response surface methodology (RSM). The effects of cathodic potential applied in the self-doping process, self-doping time, and the calcination temperature on the properties of SD-TNT films were systematically and simultaneously evaluated. Based on the current density response (*j*_c_), the FFD coupled with RSM enabled the identification of optimal experimental conditions within the investigated ranges of variables, for the synthesis of SD-TNT films. Under optimized conditions of −1.5 V applied during 480 min and calcinated at 450 °C, a current density (*j*_c_) of −0.325 mA cm^–2^ was achieved. Under these optimized conditions, EIS analyses revealed that self-doping substantially reduced the charge-transfer resistance (*R*_ct_), decreasing from 831.9 Ω for the TNT electrode to 92.35 Ω for the SD-TNT electrode. This improvement is attributed to the formation of Ti^3+^ sites and oxygen-vacancy defects, which enhance the electrochemical conductivity and facilitate electron transport across the SD-TNT electrode interface.

Finally, it should be emphasized that the present study, which employed an experimental design coupled with RSM, provided valuable insights into the factors influencing the synthesis of SD-TNT films with improved electrochemical performance.

## Supplementary Material



## Notes

Rietveld refinement parameters of TNT films; crystallite sizes of the phases; confocal Raman microscopy images of the TNT films; Raman spectra of TNT and SD-TNT films with deconvolution of the corresponding active modes; and Rietveld refinement of the X-ray diffraction patterns of the TNTs films (PDF)
