# Supplementary material for: Multivariate Optimization of Self-doped TiO2 Nanotube Synthesis for Enhanced Optical and Electronic Performance
Source: ACS Omega. 2026 Sep 28;11(40):62060–71. doi: 10.1021/acsomega.6c08217 (PMC13670801; doi:10.1021/acsomega.6c08217)
Supplement: Supplementary file 1 [file ao6c08217_si_001.pdf]

Supporting information for

Multivariate optimization of self-doped TiO_2 nanotubes synthesis for enhanced optical and electronic performance

João Vitor Martins[a][0009-0005-8816-4433], Gabrielle Sarto[a][0009-0005-3748-4525], Thiago Nogueira Marques Cervantes[a][0000-0001-6049-3343], Paulo Rogério Catarini da Silva[b][0000-0001-8593-5849], Lucio Cesar de Almeida[a,c,*][0000-0002-6395-4309]

[a] *State University of Londrina (UEL), Department of Chemistry, Laboratory of Environmental Electrochemistry (LabEA), Celso Garcia Cid Highway, PR 445, km 380, Londrina – PR, ZIP code 86055-900, Brazil.*

[b] *State University of Londrina (UEL), Department of Physics, Laboratory of thin films and materials (FILMAT), Celso Garcia Cid Highway, PR 445, km 380, Londrina – PR, ZIP code 86055-900, Brazil.*

[c] *National Institute of Alternative Technologies for Detection, Toxicological Assessment and Removal of Emerging and Radioactive Contaminants - (INCT-Datrem), Rua Professor Francisco Degni, 55, 14800-060 Araraquara, São Paulo, Brazil*

Corresponding author: ***lucioalmeida@uel.br***

Table S1 - Rietveld refinement parameters of TNT films calcined at different temperatures.

Parameters	Substrate / Ti	TNT / 450 °C	TNT / 550 °C	TNT / 650 °C
**n° Phases used**	3.000	3.000	3.000	3.000
**Zero shift / °2θ**	——	0.060	0.062	0.065
**Sample shift / nm**	–0.125	–0.200	–0.200	–0.200
**R (expected) / %**	22.72	21.95	23.52	21.50
**R (profile) / %**	23.96	19.16	20.10	18.82
**R (weighted profile) / %**	33.30	27.16	28.86	27.60
**GOF**	2.140	1.532	1.506	1.641
**d-statistic**	0.670	1.213	1.013	1.091

n° Phases used — Number of crystalline phases included in the refinement model.

Zero shift / °2θ — Constant angular correction for goniometer zero misalignment.

Sample shift / nm — Correction for specimen surface displacement from the diffractometer focal plane.

R (expected) / % — Statistically expected minimum value of R_{wp}, determined by data quality.

R (profile) / % — Unweighted reliability factor between observed and calculated profiles.

R (weighted profile) / % — Weighted reliability factor; primary indicator of refinement quality.

GOF — Goodness of fit (χ^2): R_{wp}/R ratio; values close to 1 indicate a statistically ideal fit.

d-statistic — Durbin-Watson d statistic; values close to 2 indicates no autocorrelation, while 0 positive autocorrelation and 4 negative autocorrelations.

Table S2 - Crystallite sizes of the phases at different temperatures determined by the Size-Strain Plot (SSP) and Scherrer methods.

T / °C	Crystalline phase	SSP / Å	Scherrer / Å
**450**	anatase	231.53	229.23
**550**	rutile	93.350	88.380
**550**	anatase	218.60	212.06
**650**	rutile	267.81	270.77
**650**	anatase	237.37	231.36

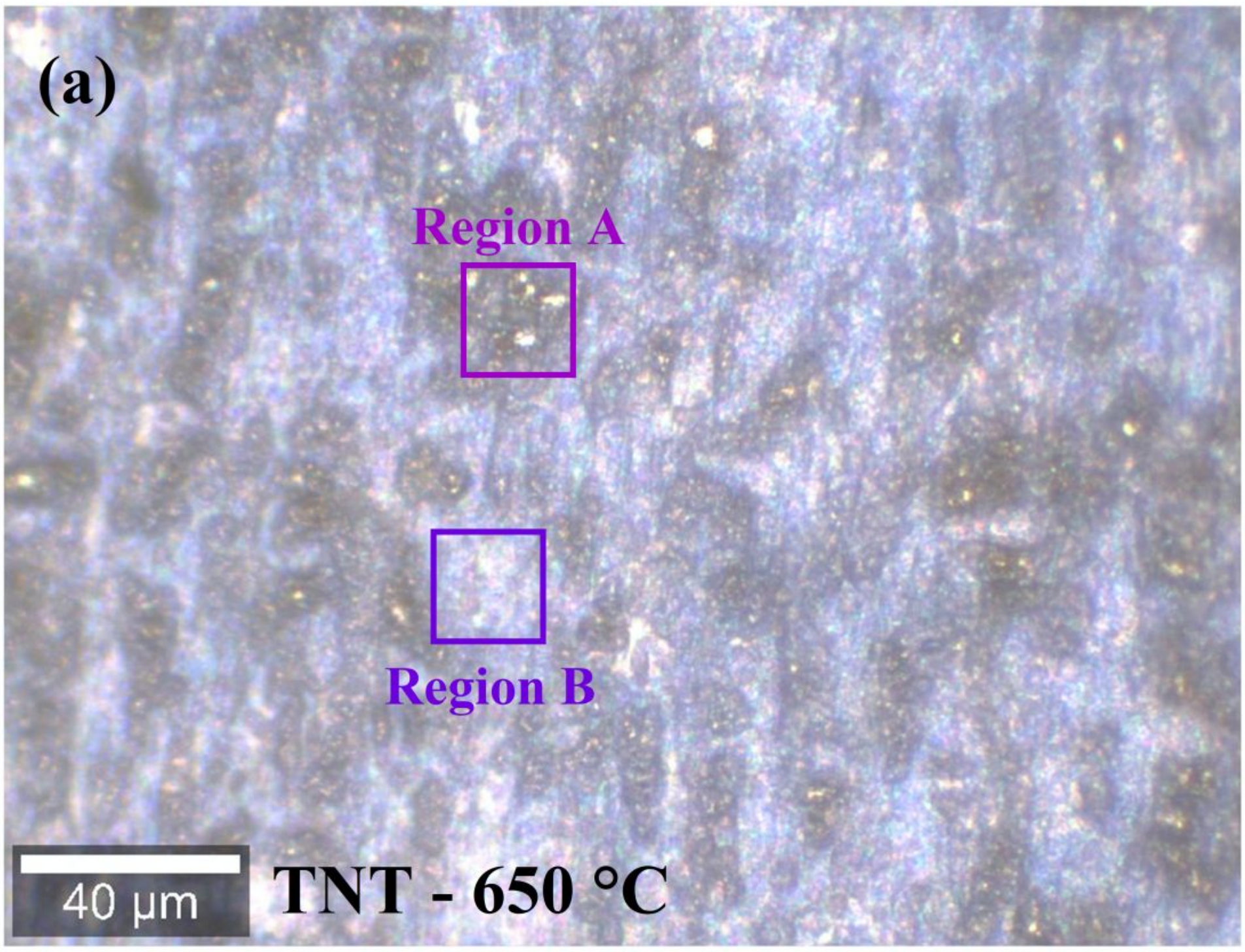
(a)
Region A
Region B
40 μm
TNT - 650 °C

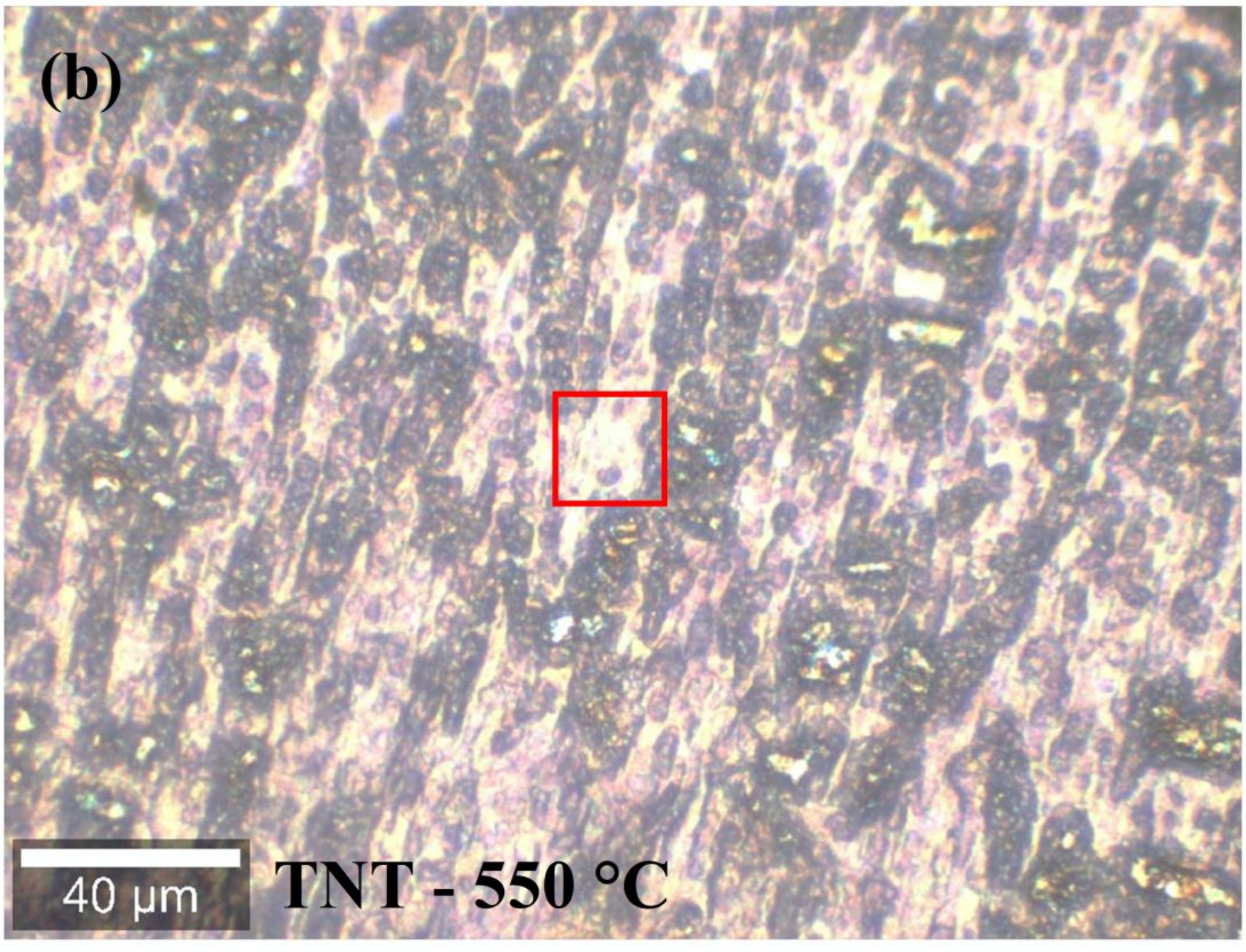
(b)
40 μm
TNT - 550 °C

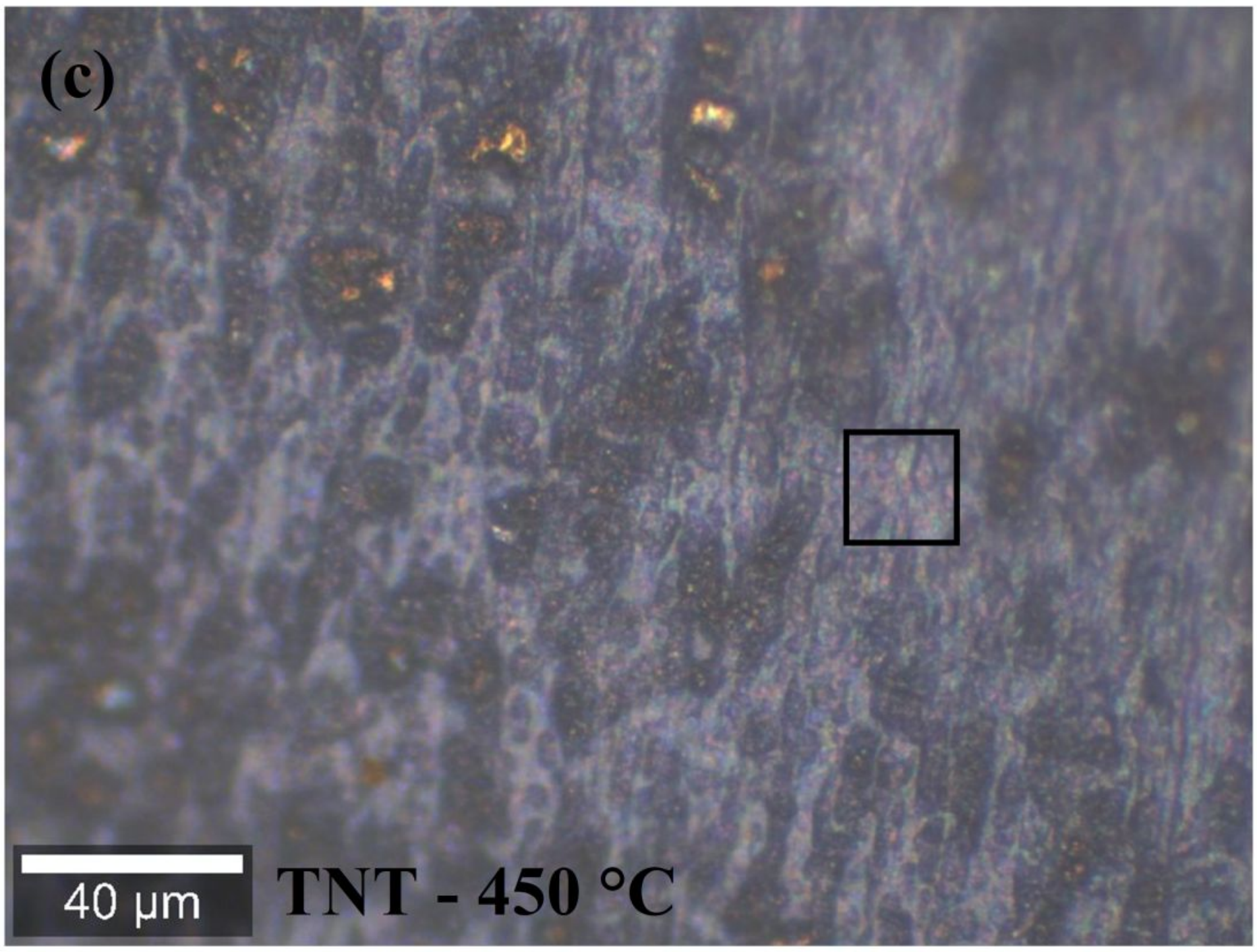
(c)
40 μm
TNT - 450 °C

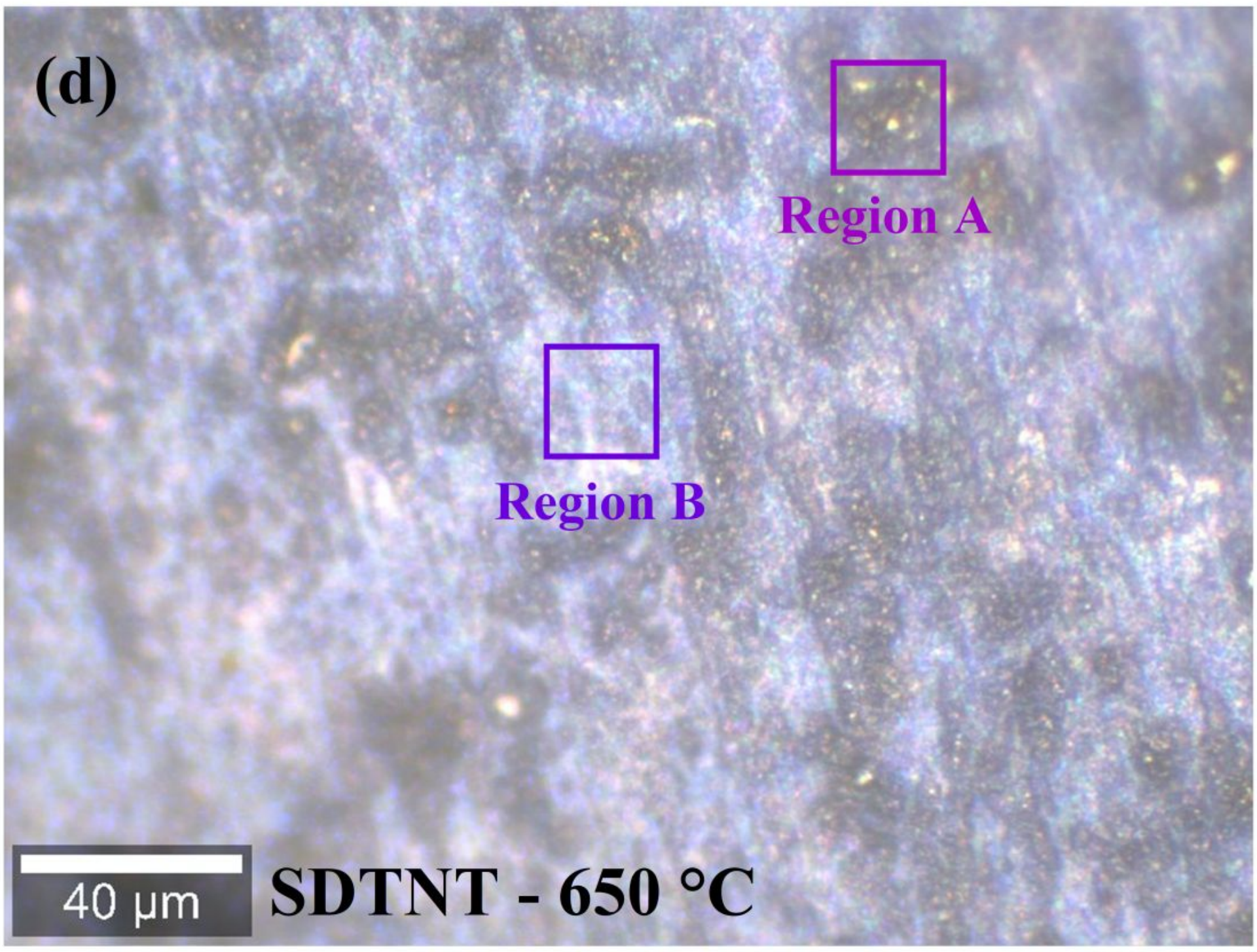
(d)
Region A
Region B
40 μm
SDTNT - 650 °C

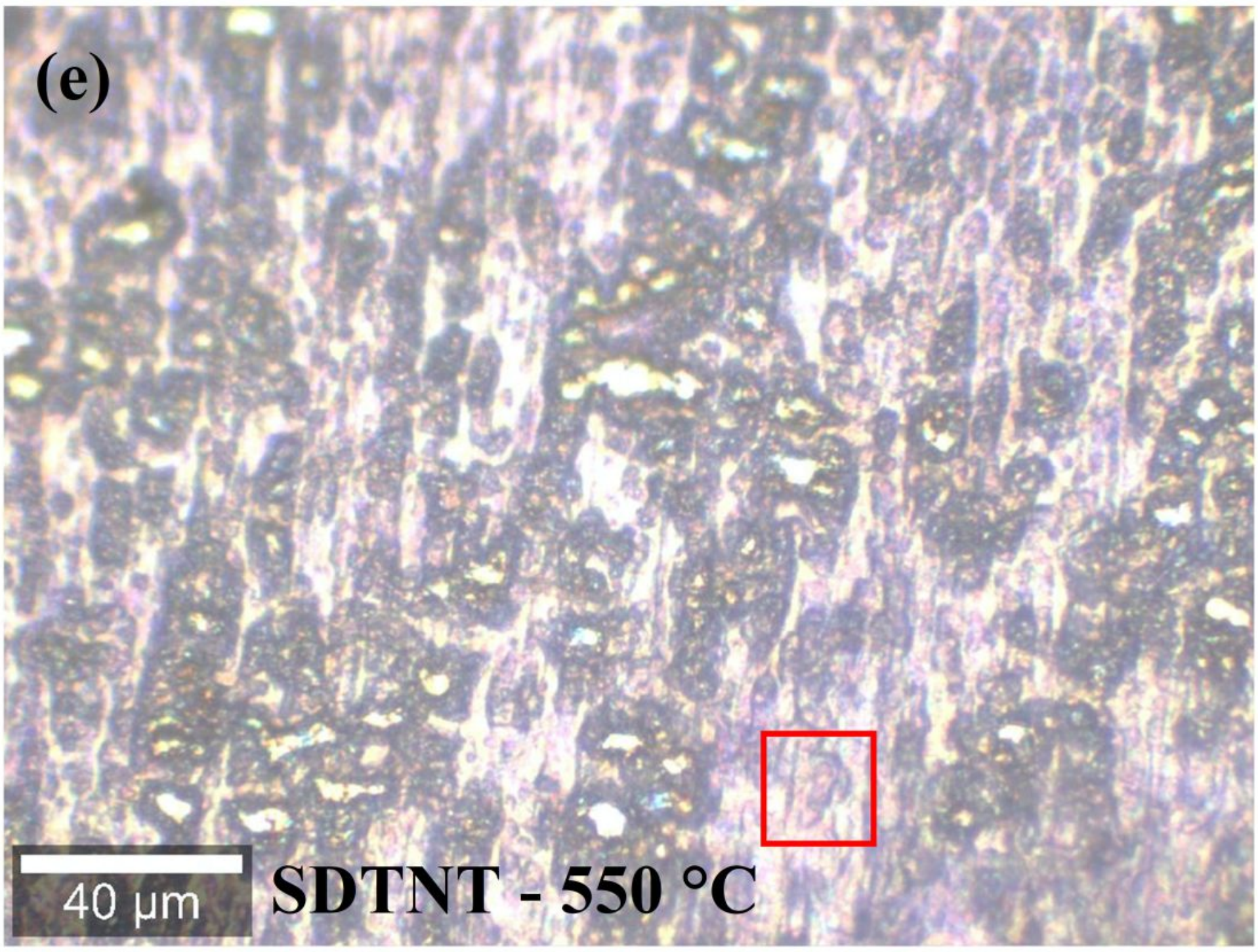
(e)
40 µm
SDTNT - 550 °C

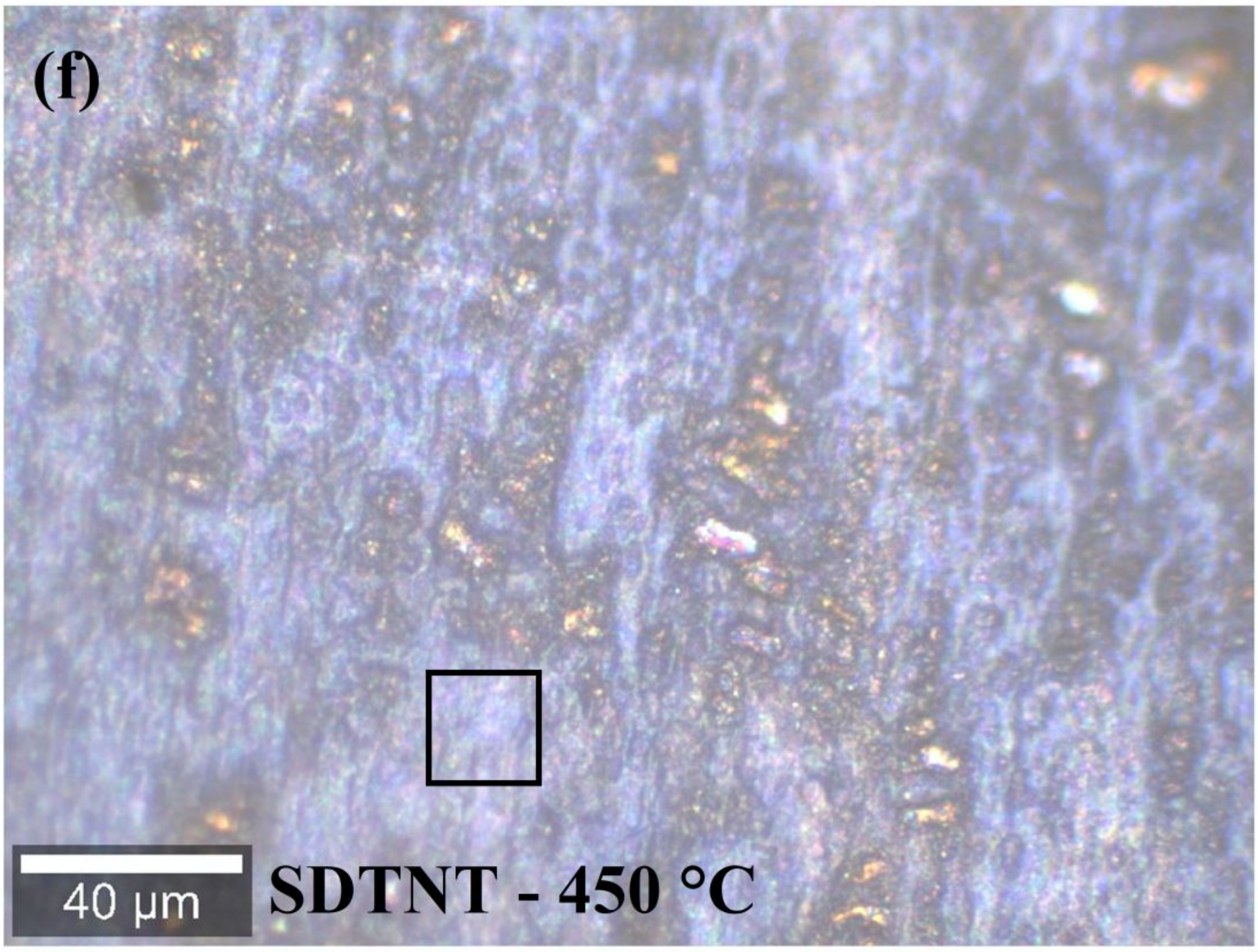
(f)
40 µm
SDTNT - 450 °C

Fig. S1- Optical images acquired by confocal Raman spectroscopy of TNT films calcined for 60 min at (a) 650 °C, (b) 550 °C, (c) 450 °C, and of SD-TNT films calcined for 60 min at (d) 650 °C, (e) 550 °C, and (f) 450 °C. All SD-TNT films were self-doped for 450 min under –1.5 V.

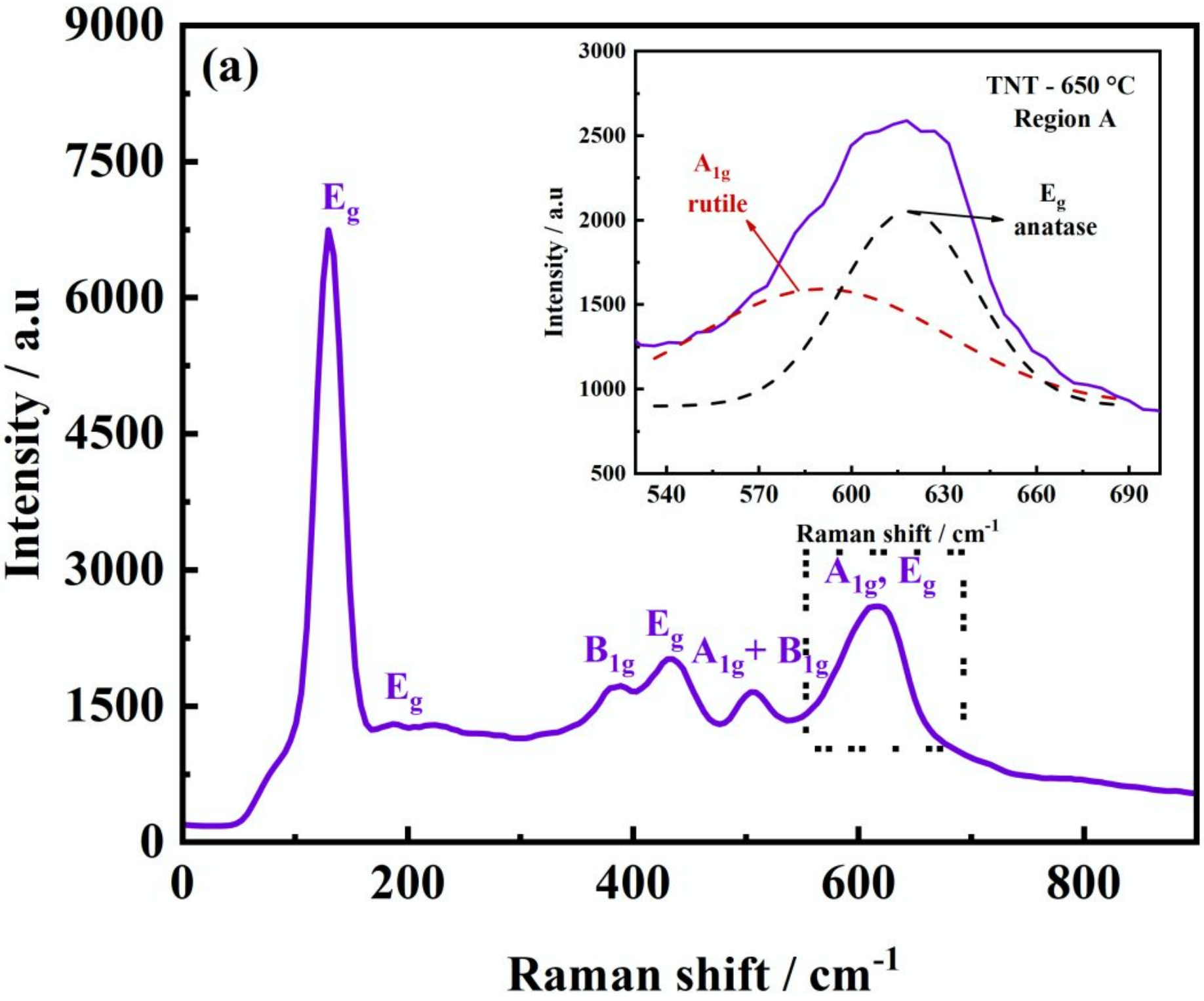

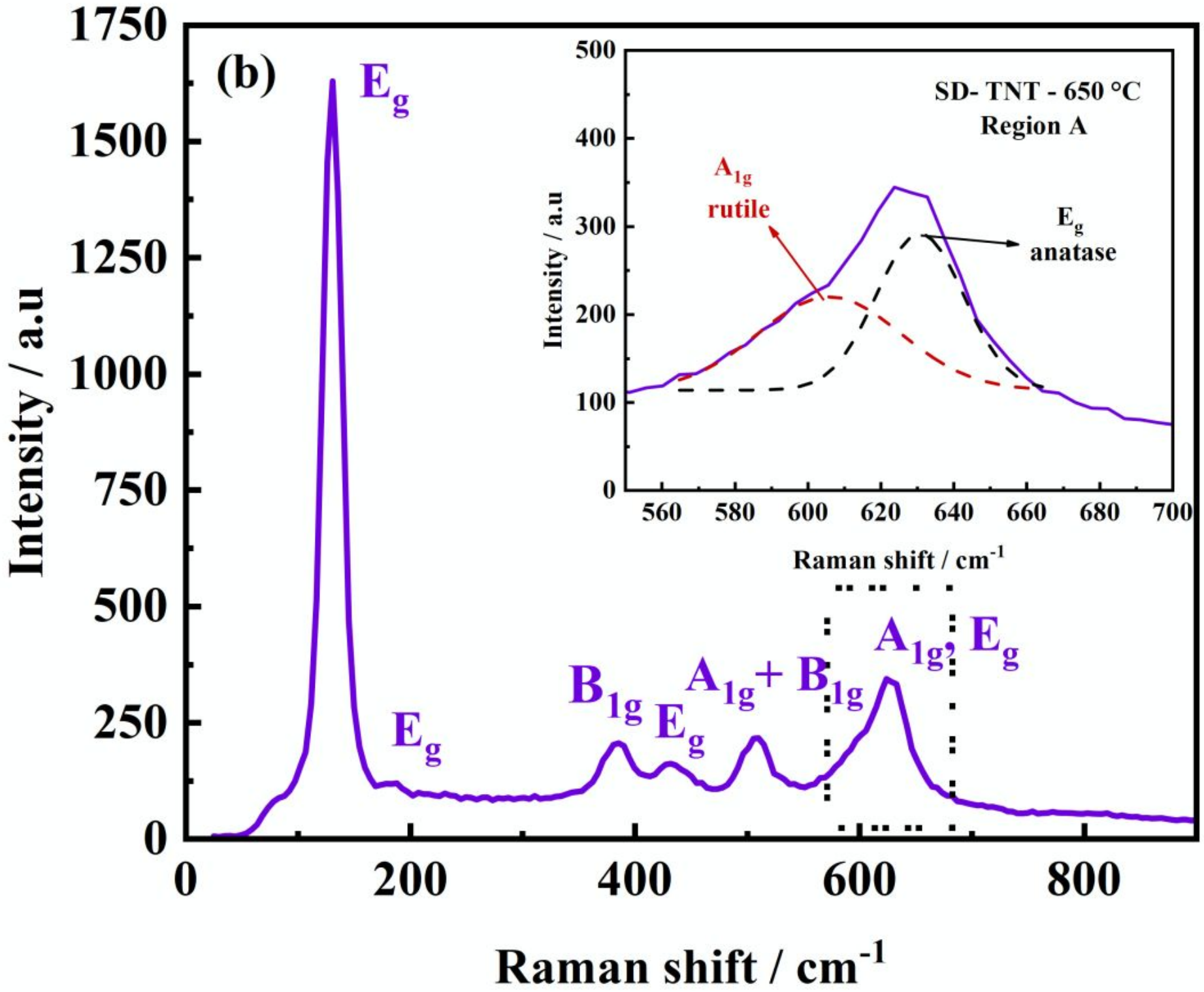

Fig. S2 – (a) Raman spectrum of the TNT film from region A, calcined for 60 min at 650 °C with deconvolution of the active mode at 612 cm^{-1} (b) Raman spectrum of the SD-TNT film from region A calcined for 60 min at 650 °C with deconvolution active mode at 634 cm^{-1}.

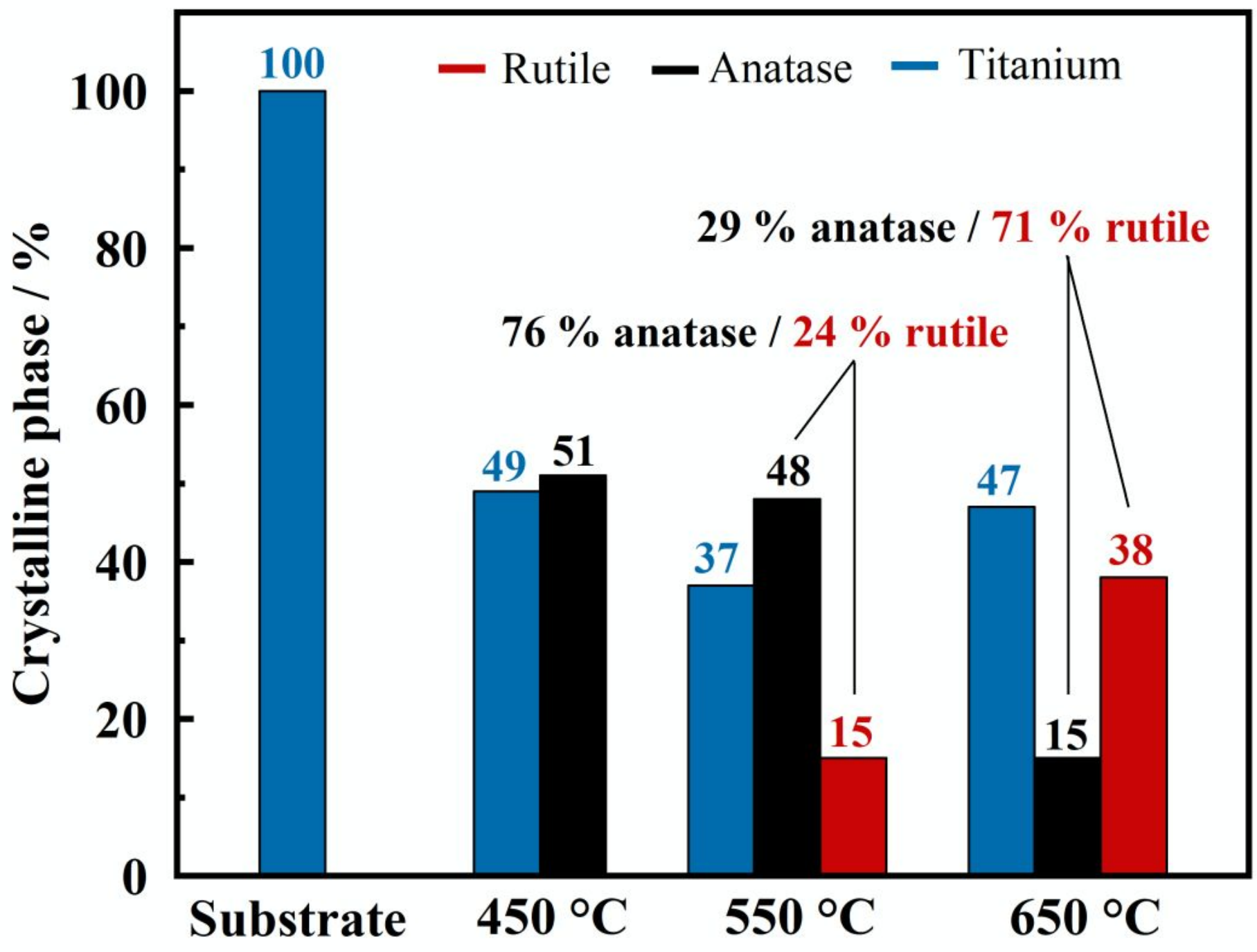

Fig. S3 - Rietveld refinement of the X-ray diffraction patterns for TNTs calcinated for 60 min at different temperatures and their relative proportions of anatase and rutile phases.

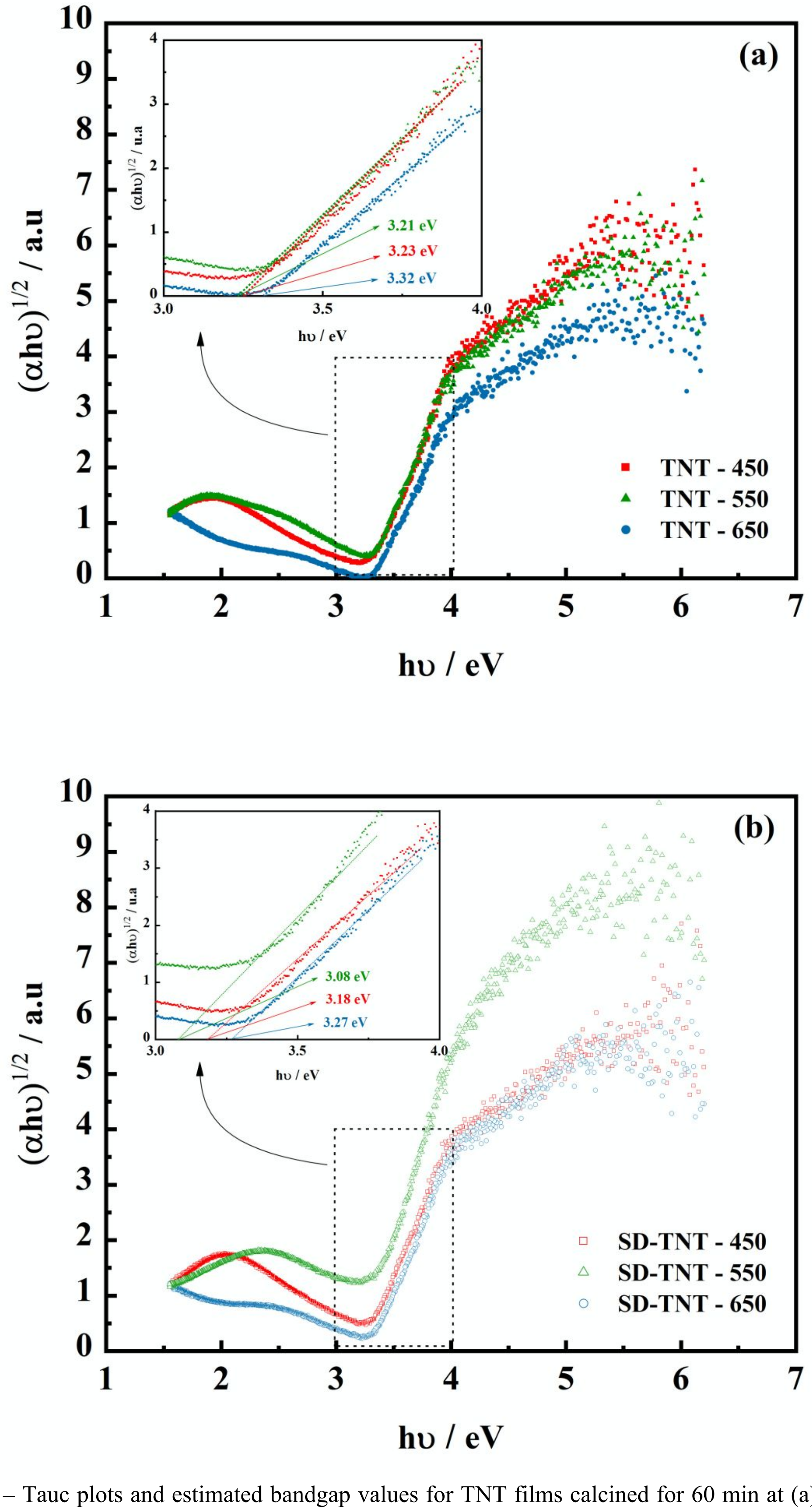

Fig. S4 – Tauc plots and estimated bandgap values for TNT films calcined for 60 min at (a) (■) 450 °C, (▲) 550 °C and (●) 650 °C, and for SD-TNT films calcined at (b) (□) 450 °C, (△) 550 °C and (○) 650 °C. All SD-TNT films were self-doped for 450 min under –1.5 V.

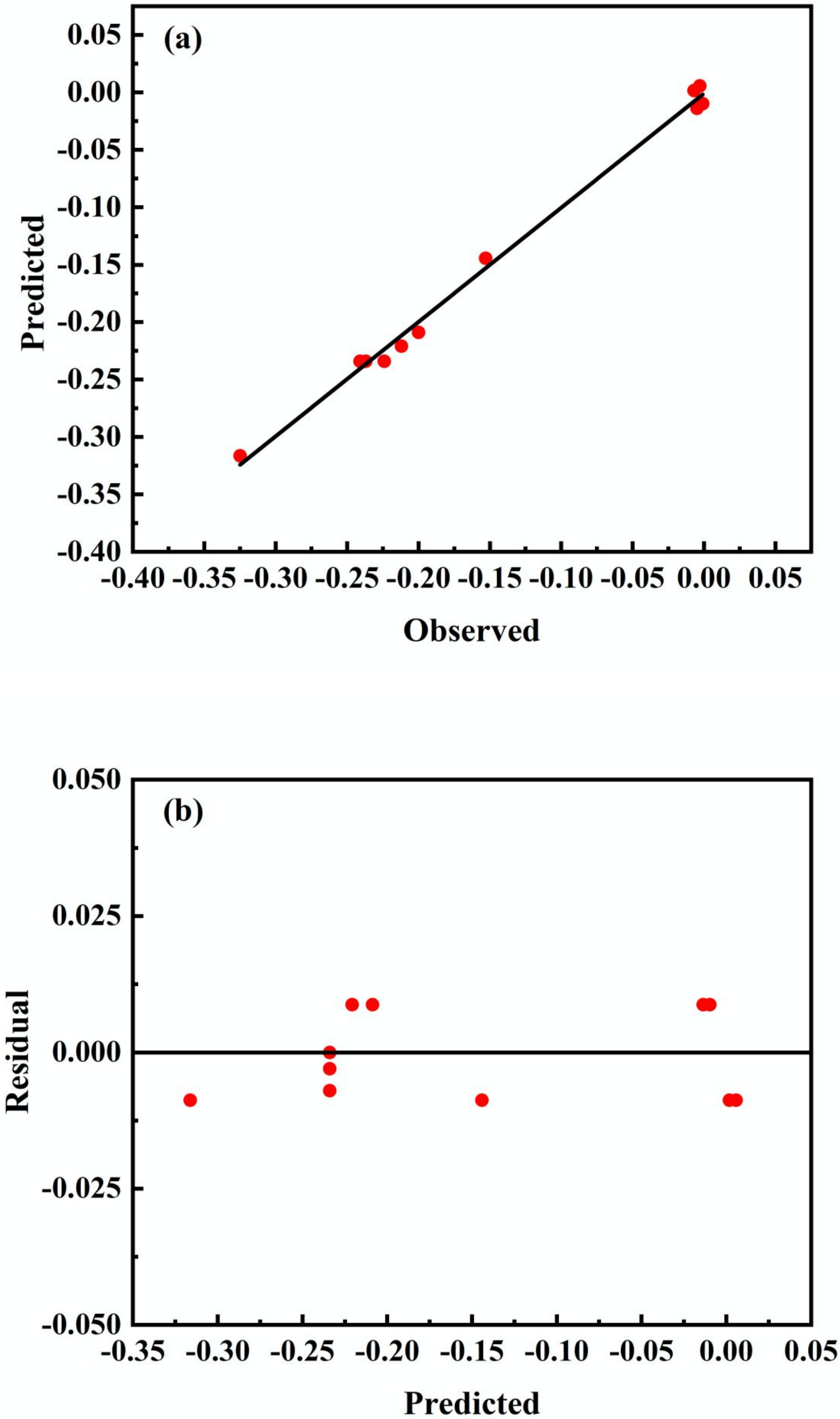

Fig. S5 – (a) Predicted-observed and (b) residual-predicted plots for the j_c response obtained using the SD-TNT films.
